# Strain Tracking with Uncertainty Quantification

**DOI:** 10.1101/2023.01.25.525531

**Published:** 2023-01-26

**Authors:** Younhun Kim, Colin J. Worby, Sawal Acharya, Lucas R. van Dijk, Daniel Alfonsetti, Zackary Gromko, Philippe Azimzadeh, Karen Dodson, Georg Gerber, Scott Hultgren, Ashlee M. Earl, Bonnie Berger, Travis E. Gibson

**Affiliations:** 1 Department of Mathematics, Massachusetts Institute of Technology, Cambridge, MA,USA; 2 Department of Pathology, Brigham and Women’s Hospital, Boston MA, USA; 3 Infectious Disease and Microbiome Program, Broad Institute, Cambridge, MA, USA; 4 Delft Bioinformatics Lab, Delft University of Technology, Delft, 2628 XE, The Netherlands; 5 Computer Science and AI Lab, Massachusetts Institute of Technology, Cambridge, MA, USA; 6 Department of Molecular Microbiology and Center for Women’s Infectious Disease Research, Washington University School of Medicine, St. Louis, MO, USA; 7 Harvard Medical School, Boston, MA USA; 8 Harvard-MIT Health Sciences and Technology, Cambridge, MA, USA

## Abstract

The ability to detect and quantify microbiota over time has a plethora of clinical, basic science, and public health applications. One of the primary means of tracking microbiota is through sequencing technologies. When the microorganism of interest is well characterized or known *a priori*, targeted sequencing is often used. In many applications, however, untargeted bulk (shotgun) sequencing is more appropriate; for instance, the tracking of infection transmission events and nucleotide variants across multiple genomic loci, or studying the role of multiple genes in a particular phenotype. Given these applications, and the observation that pathogens (e.g. *Clostridioides difficile*, *Escherichia coli*, *Salmonella enterica*) and other taxa of interest can reside at low relative abundance in the gastrointestinal tract, there is a critical need for algorithms that accurately track low-abundance taxa with strain level resolution. Here we present a sequence quality- and time-aware model, *ChronoStrain*, that introduces uncertainty quantification to gauge low-abundance species and significantly outperforms the current state-of-the-art on both real and synthetic data. ChronoStrain leverages sequences’ quality scores and the samples’ temporal information to produce a probability distribution over abundance trajectories for each strain tracked in the model. We demonstrate Chronostrain’s improved performance in capturing post-antibiotic *E. coli* strain blooms among women with recurrent urinary tract infections (UTIs) from the UTI Microbiome (UMB) Project. Other strain tracking models on the same data either show inconsistent temporal colonization or can only track consistently using very coarse groupings. In contrast, our probabilistic outputs can reveal the relationship between low-confidence strains present in the sample that cannot be reliably assigned a single reference label (either due to poor coverage or novelty) while simultaneously calling high-confidence strains that can be unambiguously assigned a label. We also include and analyze newly sequenced cultured samples from the UMB Project.

## Introduction

The human microbiome is involved in many aspects of human health and disease and exhibits a great level of diversity within and across host environments [1]. One of the most basic forms of analysis performed on any sample in a microbiome study is determining what bacteria are present and at what abundance. Although some applications call for coarser-grained taxa identification at the Operational Taxonomic Unit (OTU) or species level [2, 3], newer studies increasingly focus on more fine-grained resolution at the strain, or even Single Nucleotide Variant (SNV) level [4], including: (1) tracking *Clostridioides difficile* (*C. diff*) infection transmission events in Intensive Care Unit (ICU) patients through Single Nucleotide Polymorphism (SNP) calls [5]; (2) studying the role that individual genes play in infant gut microbial community development following antibiotic treatments [6]; and (3) detailed phylogroup analysis of *Escherichia coli* (*E. coli*) strains identified in longitudinal fecal samples from recurrent Urinary Tract Infection (rUTI) patients [7].

The process of converting bulk shotgun sequencing reads to taxa abundances usually involves some aspect of mapping or aligning reads to reference sequences [8-13]. An alternative is to perform metagenomic assembly [14], though for low-abundance taxa including most gastrointestinal pathogens of interest, this is unlikely to generate scaffolds of sufficient quality to provide strain-level insights. Unfortunately, state-of-the-art methods quantifying strain-level abundances have a multitude of shortcomings when used to track low-abundance taxa, and these shortcomings become more evident when used to study longitudinal samples. Only a select few methods report a statistic using the raw sample that can be directly interpreted as a strain’s predicted abundance. Instead, many approaches typically report *pile-up* statistics for SNPs across reference genomes or gene-specific loci [12, 15]. This then still leaves a large gap between pile-up information and the interpretation of results as strain dynamics in a longitudinal study. There are several methods that are specifically designed to de-convolve corresponding allele counts from pile-ups into abundances [9, 16]. However, per-locus pile-ups do not encode quality scores and no longer contain any information about SNV co-occurrences.

No existing method simultaneously leverages the temporal information in a longitudinal study design and the corresponding sequencing data. Indeed, base quality score information is often only used for pre-filtering low quality reads in bioinformatics pipelines [10, 17, 18]. Furthermore, no method to date utilizes the fact that multiple samples may have come from the same donor at different timepoints. Both sources of information can help overcome ambiguity when mapping or aligning reads. Notably, there are only a few methods that provide uncertainty quantification (e.g. confidence/credibility intervals) for abundance estimates. This is typically accomplished through Bayesian modeling, but previous methods take only a single sample as input and are computationally burdensome [17]. Meanwhile, recent works in other domains of computational biology have successfully demonstrated that such techniques can help overcome data sparsity and improve accuracy [19].

To address this need, we developed ChronoStrain: an *uncertainty-aware*, time-series strain tracking and abundance estimation algorithm. It is, to our knowledge, the first fully Bayesian algorithm for this problem that takes as input raw reads with associated quality score information and sample information (sample host and time of collection) to output mixtures of reference calls (estimates that incorporate all plausible candidates, and thus can be viewed as “fuzzy” probabilistic labels on a phylogenetic tree) that quantifies uncertainty in the strain abundances over time. We demonstrate the superior performance of our algorithm on synthetic, semi-synthetic, and real data. We also demonstrate the utility and interpretability of our algorithm in its application to longitudinal metagenomic data from fecal samples provided by subjects in the rUTI microbiome (UMB) study, a year long longitudinal study of women with a history of rUTI with a matched healthy cohort [7]. We additionally incorporated newly-generated culture-based sequencing data to provide higher resolution views of low abundance *E. coli*.

## Results

### Overview of ChronoStrain

**Figure 1:**
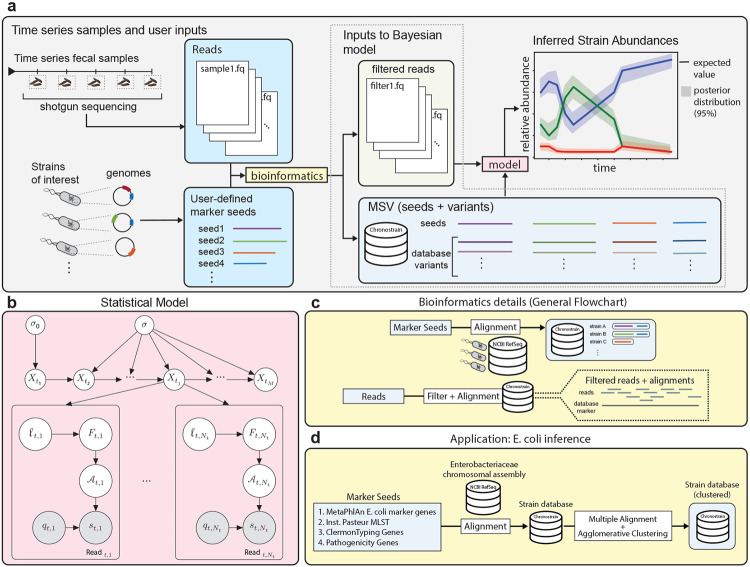
Overview of ChronoStrain **(a)** A high-level schematic of the ChronoStrain pipeline. The pipeline constructs a database from marker sequence seeds, and uses it to filter reads to be passed as input to a Bayesian inference algorithm. As output, it returns a probability distribution over trajectories. **(b)** A graphical representation of the probabilistic model (Methods - Bayesian Model) used by ChronoStrain. White nodes are latent variables, and gray nodes are observations. **(c)** The “bioinformatics” step shown in panel A first generates a database representation of strains’ marker sequences; a thorough search is performed to account for all variants or similarities. Only reads with high alignment identity to this database are kept. **(d)** For an application in tracking *E. coli* variants, we seeded our database with genes that are already widely used for defining *E. coli* strains; see Methods - Database Construction for further details.

ChronoStrain, outlined in Figure 1a, consists of three main components: (1) a custom database that is constructed from user defined marker sequences, (2) a bioinformatics step for pre-filtering and processing raw reads, and finally (3) the core Bayesian inference algorithm. The software implementing ChronoStrain is written in Python and is freely available on Github (https://github.com/gibsonlab/chronostrain). ChronoStrain takes as input three sets of files: the raw fastq files from sequencing, a metadata file containing sample host and temporal information, and fasta files containing the user-defined marker sequence *seeds*.

Each strain in our model is then defined as a collection of markers sequences that are similar in identity to our list of marker seeds. A database of these strain-marker combinations is constructed by identifying all reference sequences from NCBI RefSeq chromosomal assemblies that match a BLAST query percent identity threshold with the sequences of seeds (Figure 1c); this functionality is provided by our software. This provides the ability to specifically target user defined sequences or genes of interest. Before passing the reads onto the core inference algorithm, our software filters out reads that do not align to our custom database beyond 97.5% identity; see Figure 1c and Methods - Read Filtering for more details.

To serve as marker seeds in our analysis, we chose *E. coli* core genes from MetaPhlan [20] (v31 of the MetaPhlAn3 database [21]), Institute Pasteur’s multi-locus sequence typing (MLST) genes (https://bigsdb.pasteur.fr/), Clermont et al’s phylotyping genes [22], as well as fimH which encodes the binding region of the adhesive fiber Type 1 pili [23]. These were integrated as depicted in Figure 1d. This set of genes was chosen to help distinguish *E. coli* from other species and to provide strain level differentiation within *E. coli*. The core genes from MetaPhlan aid in distinguishing between different species, but were not necessarily designed with strain typing in mind. Thus, we also included the MLST and Clermont phylotyping genes to give further phylogroup-level resolution to the markers. fimH was added because it is currently being studied as a therapeutic target for clearing Uropathogenic *E. coli* (UPEC) [24]. With these marker sequences, our strains were defined using approximately 0.4% of a generic *E. coli* chromosome.

Our Bayesian model, for a single time series, is shown in Figure 1b. Strains are modeled using a stochastic dynamical system with the latent taxa abundance vector *X_t_j__* at time *t_j_*. Then, at each time point *t_j_*, the *i*th read is modeled as a nucleotide sequence *s_t_j_,i_* with its corresponding quality score vector *q_t_j_,i_*. The sequence *s_t_j_,i_* is modeled through the variables *F_t_j_,i_* (the source nucleotide sequence fragment of the read), *ℓ_t_j_,i_* (the random length for a sliding window along the markers that determines which fragment is measured) and ${𝒜}_{t_{j},i}$ (the fragment-to-read substitution/indel error profile). A complete description of the model can be found in Methods - Bayesian Model).

Our model is closely related to a time-series topic model [25] with an extra component that models sequencing noise. The connection is best drawn using an analogy: strains are *topics*; each abundance profile is a strain/topic mixture that produces a bag of fragments (*words*, using the language of topic models), whereby each fragment is measured with some quantifiable error per nucleotide (the bag of words is observed with typos). Both the time-series topic model and our model account for dependencies across time using a latent Gaussian process, transformed into mixture weight vectors of topics/strains via a softmax function.

To achieve scalability for our Bayesian inference (a major limitation in previous works that estimate Bayesian posteriors, see Supplemental Text §A.4), we implemented Automatic Differentiation Variational Inference (ADVI [26]) as opposed to slower sampling algorithms such as Markov chain Monte Carlo. ADVI is a gradient-based optimization method that is easily implementable on a graphics processing unit (GPU). For our model, inference was further accelerated by compressing a component of the likelihood function into a more efficiently computable one (Supplemental Text §B.1).

ChronoStrain outputs a posterior distribution over abundance *trajectories* across database strains. This is in contrast to algorithms which output point estimators (for instance, the most likely trajectory calculated via Expectation-Maximization). Specifically, in the context of longitudinal studies, ChronoStrain offers two big advantages in terms of interpretability. First, the user no longer has to stitch together outputs of an algorithm that has been run on each timepoint’s sample independently. ChronoStrain is capable of jointly modeling an entire longitudinal dataset from a single host, and thus produces abundance estimates which are more consistent throughout time than existing methods. Second, our posterior distributions offer visualizations of uncertainty that illustrate when labels are possibly ambiguous (whose mixtures form the aforementioned “fuzzy” labels) or when coverage is low.

### ChronoStrain outperforms other methods in synthetic and semi-synthetic experiments

We benchmarked ChronoStrain using *synthetic* and *semi-synthetic* read sets. For comparator methods, we have included StrainGST [15] and StrainEst [27], the top two performing alternative state-of-the-art methods. StrainGST is a *k*-mer based method that identifies taxa by performing fast mapping between *k*-merized reads and *k*-merized full reference genomes. The tool allows one to output point estimates of strain abundances over time. StrainEst is a method that deconvolves allele frequencies (generated from pile-ups against reference genomes) into abundance estimates.

**Figure 2:**
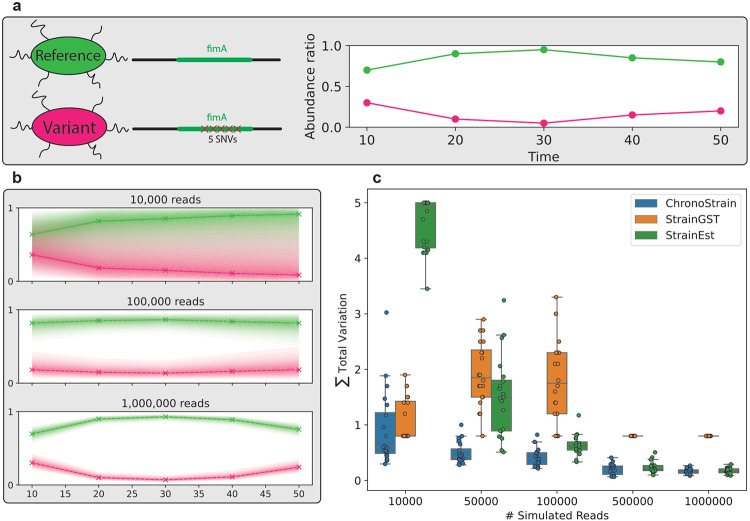
Synthetic benchmarking with two closely related strains: **(a)** To create the fully synthetic dataset, we took a reference strain and created an in silico variant from it. Reads were sampled from the depicted abundance profiles. **(b)** Example outputs of ChronoStrain as the number of synthetic reads were increased. Solid lines indicate the timepoint-wise median, and the bands of varying hues show regions of decreasing posterior confidence; the outermost bands surround the 95% credibility interval. The more reads there are the smaller the credibility interval. **(c)** A benchmark against comparator methods. The *y*-axis depicts the sum of total variation errors across the five timepoints. For ChronoStrain, we evaluate the median of this metric across the posterior, and the metric shows that this method is competitive across all coverages.

For initial benchmarking, we tested each method’s ability to track the abundance of two closely related strains (differing by only 5 nucleotides in the fimA gene) over five time points (Fig 2a). Since each relative abundance profile is a probability vector that sums to 1, we evaluated the error using the sum over time of the total variation distance (0.5 times the *ℓ*_1_ norm) to the truth; the largest possible error is the number of timepoints, which is 5. ChronoStrain outperformed StrainGST at all read depths, as shown in Fig. 2c. Even at the highest read coverage for these experiments, StrainGST levels out with a total variation error slightly below 1 and does not converge to zero even with more reads.

ChronoStrain significantly outperformed StrainEst at all read depths (Fig. 2c, p-values in Supplemental Table 1), but the gap is smaller at higher read depths. Indeed, we expect all unbiased estimators to provide similar performance as data becomes plentiful. An illustration of the uncertainty quantification capability of ChronoStrain is given in Figure 2b, showing how the credible intervals of the trajectories shrinks as the read depth increases. Full implementation details for the synthetic experiments can be found in Methods - Fully Synthetic Benchmark.

**Figure 3:**
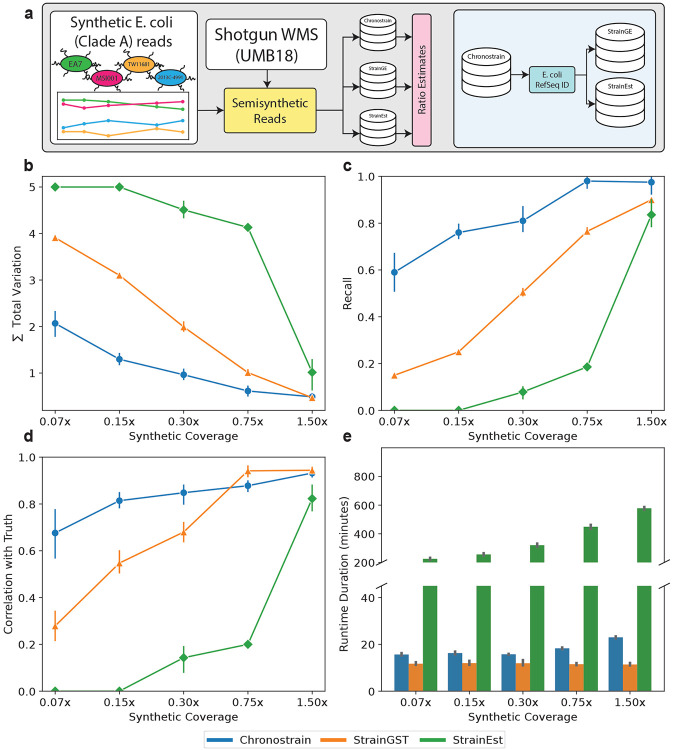
Semi-synthetic benchmarking with simulated *E. coli* reads appended to real data. **(a)** We combined synthetic reads from hand-picked *E. coli* reference genomes, and combined it with real data from the UMB dataset. For the sake of benchmarking, we initialized comparator methods’ databases with the same collection of strains. **(b)** A benchmark of the total variation error in abundance profiles for each method shows that ChronoStrain is competitive across the board. **(c)** The recall ratio, measuring the fraction of true positives (refer to Online Methods for how detection was defined in ChronoStrain.) **(d)** Plotting the time-averaged Spearman correlation shows the rate at which the methods learn the ranking of strains, sorted by abundance. A higher value is better, since correlation is measured with respect to the ground truth. **(e)** In terms of runtime, StrainGST was fastest, followed closely by ChronoStrain. ChronoStrain scales roughly linearly with the number of reads that hit the database. All x-axis values report coverage using only simulated reads, and does not count the real data (roughly 19,000,000 reads per timepoint).

Moving on to a more challenging and realistic benchmark, inspired by [15], we created a semi-synthetic dataset by simulating reads from four reference genomes (EA7, MSI001, TW11681, 2013C-4991) all within *E. coli* Phylogroup A as identified by the ClermonTyping [22] software, and mixing these *in silico* reads with the real metagenomic reads from the first five fecal samples collected from one participant in the UMB study (UMB18, Figure 3a). While strains from phylogroups B2 and D were detected among the UMB18 samples, none from Phylogroup A were identified using any of the methods discussed in this section. With this setup, we have a ground truth subset of four strains — whole-genome average nucleotide identity (ANI) ranging from 98.5% to 99.5% — where the algorithms must also contend with the fact that there are other reads originating from *E. coli*. We benchmarked the algorithms’ performances using four metrics: abundance estimation error (measured via the sum of total variation, Figure 3b), classification error (recall rate for calling the *in silico* strain IDs, Figure 3c), the spearman correlation to the ground truth (Figure 3d), and runtime (Figure 3e). For more details on how these metrics were computed, see Methods - Semi-synthetic Benchmark.

ChronoStrain significantly outperforms StrainEst in all metrics and requires an order of magnitude less runtime. At low coverage (0.07x-0.15x), StrainEst has zero recall and zero correlation with the ground truth. ChronoStrain outperforms StrainGST in terms of total variation until 1.50x coverage, recall for all coverage levels, and Spearman correlation until 0.75x coverage; *p*-values testing for the differences in these metrics are provided in Supplemental Table 2. As with the fully synthetic data, we expect StrainEst’s performance to be indistinguishable from ChronoStrain with sufficiently high read depth. In these examples, ChronoStrain’s runtime varies from 120%~200% that of StrainGST. StrainGST’s runtime is theoretically linear in read depth (processing reads into *k*-mers), but since it is already fast with respect to the real reads, there is almost no visible difference when varying synthetic read coverage (the simulated reads make up less than 1% of the full dataset). StrainGST’s speed comes from its use of sparsified *k*-mer counts stored in hash tables, requiring storage of a sublinear number of *k*-mers, incurring a *O*(1)-time lookup. StrainEst, on the other hand, is slow because it solves a large Lasso regression problem. ChronoStrain’s runtime depends roughly linearly (due to the sparsification step as seen in Methods - Target Posterior Approximation) on the number of reads that map to our database. Furthermore, half of our runtime was spent running bowtie2 [28], which was chosen to create a fairer comparison to StrainEst, which also uses the tool but could have been substituted with a more scalable alternative (e.g. CORA [29]).

### Analysis of UMB dataset with ChronoStrain provides interpretable results with more consistent correlations over time

The UMB project [7] monitored 31 women in two cohorts, “rUTI” (multiple UTIs in past year) and “healthy” (no recent history of UTI), over the course of a full year. Each participant provided a stool sample once a month, with outgrowth cultures grown from rectal and urine samples taken at the first month for all participants. For those participants who were clinically diagnosed with a UTI, additional urine samples and outgrowth cultures were taken on the days of diagnoses when possible. Beyond this, metadata about participants’ self-reported dates of last known antibiotic administration and the dates of infection are available. In addition to the original samples, we have added a new data modality. For specific samples from the rUTI cohort for which blooms were estimated by StrainGST, cultures from stool samples plated on MacConkey agar (favoring Gram-negative bacteria including *E. coli*) were sequenced.

**Figure 4:**
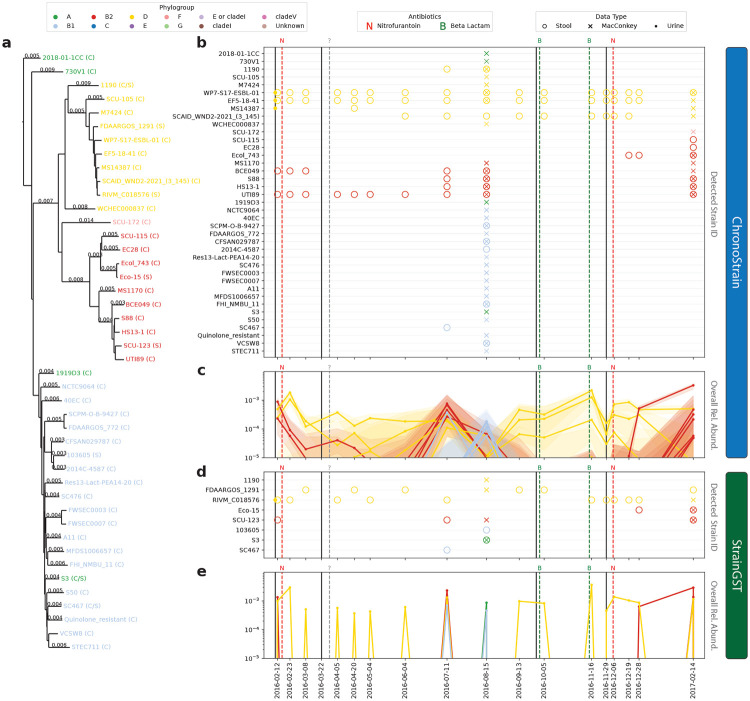
A visualization of ChronoStrain’s and StrainGST’s outputs for UMB18, a rUTI-positive participant. **(a)** A phylogenetic sub-tree of strains detected in either method, estimated using whole-genome Mash distances. Leaves are colored by phylogroup; labels with C(hronostrain) and/or S(trainGST) indicate whether the leaf was a representative in the respective methods’ databases. **(b,d)** A scatterplot of strain detections across timeseries for the two respective methods. Different markers indicate sample modality (Stool, MacConkey culture from stool, urine). Solid vertical lines indicate dates of UTI diagnoses, dotted lines indicate self-reported, last-known dates of antibiotic administration. **(c,e)** Plots of estimated *overall* relative abundances in stool.

We applied ChronoStrain and StrainGST to all 31 time-series in the UMB study (Supplemental Figures) with results for UMB participant 18 shown in Figure 4.^1^ Urine samples and MacConkey cultures were also analyzed, but independently of the fecal time series. To assist with interpretability, we encoded strains with phylogroup annotations; this provides a coarsened picture of both algorithms’ outputs. Overall, ChronoStrain identified more taxa marked as detected (open circle for fecal samples, Figure 4b,d) than StrainGST. This is to be expected, as StrainGST is required to estimate a *single* most likely strain for each *k*-mer within a read, where as ChronoStrain provides a posterior distribution over the *plausible* strains associated with each read. For details on how posterior distributions from ChronoStrain are used to decide when strains are detected see Methods - Detection Classifier.

Interpreting the output of ChronoStrain (Figure 4b,c) we see that the initial infection most likely came from a Phylogroup D strain (closed circles on the solid line just before 2016–02–12). After multiple rounds of antibiotics, the phylogroup D strain(s) are no longer detectable in the urine but still persist in the GIT. Two of the Phylogroup D strains are consistently detected in the GIT. The representative strains (WP7–S17–ESBL–01 and EF5–18–41) From phylogroup D are called in the first urine sample (closed circles) just before 2016–02–12 and are both marked as detected in 16 of the 17 fecal samples (open circles). Another prominent phylogroup in the time series is B2 which shows differing responses to the antibiotics. The initial dose of nitrofurantoin and the unknown antibiotic reported by the participant shortly after fail to clear the B2 phylogroup from the GIT with B2 references strains identified in the first 9 fecal samples, the most consistent of which is reference label UTI89. Around the time of the third and fourth round of antibiotics, which were beta-lactam, all the B2 reference strains drop well below a median relative abundance of 10^−5^. Then shortly after the fifth round of antibiotics, returning to nitrofurantoin on 2016–11–29, the model identifies the rapid growth of B2 strains. For the later timepoints, the model detects B2 strains that were present earlier and strains within a different subgroup of B2 (that includes Ecol_743) that was undetected previously. The reference strain Ecol_743 was also detected in the enriched sample on 2017-02-14, which adds evidence to support the emergence of a new B2 strain at the end of the time-series. These results suggest beta-lactam has an improved ability to suppress B2 in the GIT over nitrofurantoin.

Interpreting the output of StrainGST (Figure 4d,e) one sees inconsistent calls of Phylogroup D strains over time. Within this phylogroup, the strains FDAARGOS_129 and RIVM_C018576 are called in a mutually exclusive fashion for the fecal samples. The urine sample just before 2016-02-12 identifies RIVM_C018576 (closed yellow circle) as detected, but for the MacConkey-enriched sample on 2016–08–15 two different Phylogroup D labels are marked as detected (yellow x’s for FDAARGOS_129 and 1190). Then, for the enriched outgrowth sample on 2017–02–14, strain RIVM_C018576 is called; this is consistent with the early urine sample, but the regularly sequenced fecal sample on the same day (open yellow circle) marks FDAARGOS_1291 as detected. Phylogroup B2, which was largely undetected by StrainGST in the first half of UMB18’s time-series, was called in the enriched sample on 2016–08–15. Overall, StrainGST exhibits inconsistent strain calls over time when compared to ChronoStrain’s output. This makes it difficult to evaluate the sensitivity of different phylogroups to antibiotics or to determine the presence of new strains from a bloom. Furthermore, the lack of a credibility (or confidence) interval hampers the interpretability of StrainGST.

**Figure 5:**
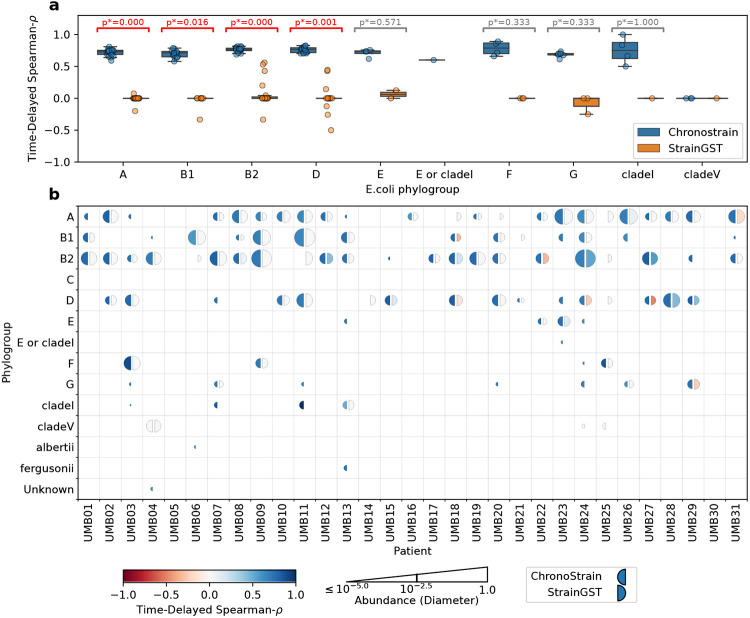
ChronoStrain provides time-consistent predictions. To quantify the improvement in the consistency across time compared to state-of-the-art predictions, we computed the time-delayed, phylogroup-specific Spearman-*ρ* for all participants. For ChronoStrain, we consider the median coherence across posterior samples. **(a)** By aggregating across all participants, we performed two-sided Wilcoxon signed-rank tests for each phylogroup. Significance was determined via Benjamini-Hochberg (BH) correction at a 5% false discovery rate. Displayed *p*-values are also BH-adjusted. This result suggests that StrainGST’s output strains from phylogroups A, B1, B2, D are inconsistent across time **(b)** For each phylogroup, we plotted *ρ* and maximal abundance estimates for each participant. The size of each semicircle depicts a log-scale abundance level for the corresponding method, relative to the entire sample (details in Supplement), while the color depicts the Spearman coherence. Both algorithms largely report similar abundances for the most dominant phylogroups, but ChronoStrain reported D, F and G more often than StrainGST.

In order to quantify ChronoStrain’s qualitatively observed increased temporal consistency in strain detection over StrainGST, we computed the time-lagged Spearman correlation (the abundance rank correlation between adjacent timepoints) for all strains across all participants in the UMB study Figure 5. The microbiome has been shown to have long-term stability of strains [30], and has been demonstrated to quickly reach (within weeks) an equilibrium after perturbation (e.g. antibiotics) which is stable until another perturbation is introduced [31, 32]. Using temporal correlation to measure stability [30] and based on these prior studies, we expect the UMB time-series to have positive temporal correlation. For the top four most abundant phylogroups (A, B1, B2, D), ChronoStrain produced a significantly higher time-lagged correlation coefficient (approximately 0.75) when compared to StrainGST’s outputs (approximately 0 correlation and sometimes even negative), Figure 5a. For the next four most abundant phylogroups (E,F,G, clade I), ChronoStrain again had higher correlation coefficients than StrainGST (0.75 vs 0), but these were not significant due to the lack of UMB participants with those strains detected.

## Discussion

There are two major differences between ChronoStrain and comparable methods. First, ChronoStrain learns a distribution over strain abundances, while the others provide a single point estimate. Second, ChronoStrain is fully Bayesian: it explicitly propagates measurement uncertainty and temporal information between samples, while other methods do not account for either source of information. With these differences, ChronoStrain’s performance under semi-synthetic benchmarking was competitive with StrainGST and StrainEst while using 1/10th and 1/20th the reads respectively (Figure 3c). These kinds of performance improvements translate to nontrivial savings in terms of one’s experimental budget.

These differences also allow for a qualitatively different approach to interpreting results with ChronoStrain, making it possible to directly visualize uncertainty through the plotting of credible intervals or to possibly use the entire posterior distribution in downstream analyses. The fact that a posterior distribution is learned for this model also has a direct advantage over the other methods by removing the need for any initial steps of agglomerating reference genomes. For StrainGST one does not estimate taxa abundances for all species in the database directly. It instead agglomerates similar strains together and then outputs abundances for each agglomeration. Since StrainGST only performs point estimation, the output from the model is very sensitive to this threshold. If the agglomeration is too fine-grained, there is no consistency over time with respect to which strains are detected. By making the agglomerations coarser, one will likely have more consistent calls over time but lose the granularity required to distinguish strains; this is also discussed by the StrainGST authors [15]. To provide a direct comparison, we agglomerated the reference genomes in our UMB analysis to the same ~99.7% ANI [7] for all methods even though this is not theoretically required for ChronoStrain.

Recall that ChronoStrain defines a strain as only those bases associated with marker sequence seeds, and in this study, we define a strain using 0.4% of a generic *E. coli* chromosome. It may be surprising/counter-intuitive that these performance improvements are being achieved with far shorter reference sequences (than e.g. the reference genome — see Supplemental Text §A.1). However, by targeting discriminatory regions of the genome, one is able to focus the model on the shorter but potentially more informative sequences [33]. Our definition of “strain” in terms of what gene variants are present or absent does have distinct advantages in several settings; it allows the user to define specific genes, cassettes and/or plasmids of interest, or to potentially track functional capabilities as apposed to taxa.

The primary shortcoming of our model is that it operates with a reference database in conjunction with marker sequence seeds. Moving beyond this it would be useful to adopt a methodology for *de novo* assembly of marker sequences. Our model could also be used to overcome ambiguity for scenarios where the forward and reverse reads overlap, which occurs in 16Sv4 Amplicon sequencing. In future work we also plan to address the use of long reads as these technologies are becoming more relevant in metagenomic studies. Even with the increased accuracy that these methods are now achieving one is still expected to have approximately 27 errors per read (for HiFi: ~99.8% accuracy with 13.5 kilobase read length on average [34]). ChronoStrain’s ability to leverage quality scores and additional sample information would be ideal for overcoming ambiguity with discrepant bases when trying to construct contigs.

## Conclusion

Uncertainty quantification is an often overlooked concept in computational biology. Our Method, ChronoStrain, incorporates uncertainty and time-awareness throughout its Bayesian probability model and exhibits significant performance improvements over current state-of-the-art. This allows for a more interpretable representation of how raw reads were likely to come from different genomes, and how that impacts our ability to estimate their abundances over time. We believe these more interpretable results will have direct impacts on biological insights, while the performance improvements will have nontrivial implications for the performance-to-cost ratio.

## Online Methods

### Overview of ChronoStrain

ChronoStrain is a Bayesian inference algorithm that learns the posterior distribution of bacterial strains’ relative abundances from metagenomic shotgun reads, by focusing on a subset of them which map to particular sections of the genome (e.g. marker sequences). Precise details of our model and its implementation is given in the Supplement; here we only give the high-level overview and explain key modeling decisions.

As input, one provides a database 𝒮 of strains and their marker sequences, a list of timepoints 𝒯 and each timepoint *t*’s collection of *N_t_* corresponding reads. For each timepoint t∈𝒯 and $i\in [N_{t}]$, each observed read *r_t,i_* = (*s_t,i_*, *q_t,i_*) is modeled as a (nucleotide sequence, phred quality score vector) pair.

The core machinery of the algorithm is based on a Bayesian model describing the joint distribution of $(X_{t},R_{t}{)}_{t\in 𝒯}$, where *X_t_* is a latent representation of the unobserved abundance profile at time *t*, and *R_t_* is the sub-collection of reads of size *N_t_* which align to our database. The latent representation $(X_{t}{)}_{t\in 𝒯}$ is modeled using a standard Gaussian random walk (Wiener process [35]), and is linked to the abundance trajectory by a sigmoidal function, resembling dynamic topic models [25]. Each read in *R_t_* is modeled as being conditionally independent, given $X\;=\;(X_{t}{)}_{t\in 𝒯}$. For the reads, we use the Phred model with random insertions and deletions, together with a model that considers a random choice of a position on the genome from the latent population that the read is measuring. We believe that the latter part is especially important for strain-level resolution, since we care about single-nucleotide variants of genes and thus one *must* consider the eventuality that reads will be mapped to multiple genomes.

ChronoStrain performs variational inference to approximate the posterior distribution *p*(*X* ∣ *R*). We note that the model found in this paper is specifically tailored for short Illumina reads; we will point out exactly where this assumption is encoded.

The main difficulty for this task is in making the resulting algorithm scale efficiently with model size, which depends on the marker lengths, strains, and reads. Indeed, if implemented naively, practical computers would not have the required memory to store the full probabilistic model (easily taking up to hundreds of gigabytes for the database that we used), even if just learning *E. coli* ratios. Our solution to scalability involves a heuristic sparsification of the data likelihood function (mathematically derived in Supplemental Text §B.1). We show, using provided benchmarks on synthetic and semi-synthetic data (Figure 3), that it works well in practice.

### Bayesian Model

First, we model the unobserved abundances using latent representations $X_{t_{1}},\ldots ,X_{t_{T}}\in R^{𝒮}$. The *X_t_*’s are timepoint-wise observations of a time-rescaled Weiner process (a non-stationary Gaussian Process):

$$\;X_{t_{1}}∼𝒩(0,\sigma_{0}^{2}I)\;X_{t_{j}}\;|\;X_{t_{j-1}}∼𝒩(X_{t_{j-1}},\sigma^{2}(t_{j}-t_{j-1})I).$$

where the scalar variance parameters $\sigma_{0}^{2}$, $\sigma^{2}$ are given by two independent instances of Jeffrey’s (improper) prior [36] for the variance of a Gaussian with known mean:

$$p(\sigma_{0})\propto \frac{1}{\sigma_{0}}\;\;\;\text{and}\;\;\;p(\sigma )\propto \frac{1}{\sigma }$$

This prior was chosen to fulfill the role of a “non-informative” prior, and for its transformational invariance property. For users of our method, this means that the choice of measurement units of time – whether it is minutes, hours or days – does not matter. To transform these realvalued vectors into relative abundances, we take *Y_t_* = softmax(*X_t_*), so that $Y_{t}\;\in \;\Delta^{𝒮-1}$, the 𝒮-component probability simplex. This particular functional composition is seen in other models in machine learning literature, such as continuous-time dynamic topic models [25, 37].

Next, we model the reads in two steps: the random position of the reads’ source fragments and then the sequencing noise. We model each read *r_t,i_* as being a noisy measurement of a single randomly chosen *fragment f_t,i_*. For paired-end reads, we modeled each end independently (only about 2% of all aligned, filtered mate pairs map to the same marker). In our model, a “fragment” is a substring of a marker sequence. The primary assumption that we will make here is that each read’s source fragment overlaps with *some* marker in the database, thus necessitating a filtering step as described in Methods. Conditioned on the *Y_t_*’s, we model the (timepoint *t*, index *i*) read *r_t,i_* independent from all other reads as described below.

First, we introduce a few definitions. Allowing each marker sequence of strain genome *s* to be padded with *β* “empty” nucleotides on both ends (*β* is a parameter of the model), let ${𝒲}_{s}^{\left(\ell \right)}$ be the collection of all length-*ℓ*, position-aware sliding window of markers of *s* for positive integers *ℓ*. We say that $w\in {𝒲}_{s}^{\left(\ell \right)}$ induces *f* if *f* is the string obtained from *w* by removing all padded bases; in particular, *f* is always at most as long as *w* (∣*f* ∣ < ∣*w*∣). For this work, we take *β* = 0.5 × (median read length), so that each marker fragment (roughly) represents half of each aligned read. Let $\Sigma_{s}^{\left(\ell \right)}$ denote the set of all substrings induced by each $w\in {𝒲}_{s}^{\left(\ell \right)}$. Finally, let $n_{f,s}^{\ell }=|\{w\in {𝒲}_{s}^{\left(\ell \right)}\;:w\;\text{induces}\;f\}|$ be the number of times *f* is induced, and let $n_{s}^{\left(\ell \right)}=\sum_{f\in \Sigma_{s}^{\ell }}n_{f,s}^{\left(\ell \right)}$ be its sum across all *f*.

Using the above definitions, we describe the fragment model. For each read *r_t,i_* let *ℓ_t,i_* be NegBinomial(*r*_0_,*p*_0_)-distributed. We model *f_t,i_* as being sampled proportional to the frequency at which it is represented in the population at time *t*. More precisely (dropping the subscripts *t*, *i* to make it easier to read):

(1) $$p(f\;|\;Y_{t},\ell )\propto \sum_{s\in 𝒮}Y_{t}(s)n_{f,s}^{\left(\ell \right)}$$

Note that this proportionality represents a normalization across all fragments *f*, and the normalization denominator $\sum_{f}\sum_{\hat{s}}Y_{t}(s)n_{f,\hat{s}}^{\left(\ell \right)}=\sum_{\hat{s}\in 𝒮}Y_{t}(\hat{s})n_{\hat{s}}^{\left(\ell \right)}$ is a function of *Y_t_* and *ℓ*. Algorithmically, a certain approximation of this (Supplemental Text §B.1.2) results in a natural correction for strains whose markers are over-represented in the database. As an aside, we remark that this is precisely the part of our method specially tailored for short reads. When operating on long reads (~10-25 kb or longer), the approximation fails to hold and thus our algorithm must be adapted to a different strategy.

Each read *r_t,i_* = (*s_t,i_*, *q_t,i_*) ∈ *R_t_* is modeled in the following way, treating *q_t,i_* as a fixed observation. Conditioned on *f_t,i_*, we model the per-base sequencing error given by indels and substitutions, which we notationally represent using the language of alignments. We write

$${𝒜}_{t,i}\;|\;f_{t,i}∼\text{PHRED}\;\text{WITHINDELS}(f_{t,i},q_{t,i})$$

where ${𝒜}_{t,i}$ is an arbitrary alignments of *s_t,i_* to *f_t,i_*, represented bya 2 × *K* array of nucleotides and gap symbols ‘−’.

We temporarily drop the subscripts for exposition. The PhredWithIndels(*f*, *q*) distribution is supported over *feasible* alignments (e.g. alignments in the theoretical search space of the Needleman-Wunsch dynamic programming algorithm [38]). For example, 𝒜 must satisfy max(∣*s*∣, ∣*f* ∣) ≤ K ≤ ∣*s*∣ + ∣*f* ∣ and (# matches) + (# mismatches) + (# deletions) = ∣*f*∣. Its likelihood function is given by a formula assuming fixed indel error rates *ε*_ins_ and *ε*_del_ (Supplemental Text §B.2) and the standard phred score model:

$$p(𝒜\;|\;f;q)\;\;=(\varepsilon_{\text{del}}{)}^{\left(\#\;\text{deletions}\right)}(\varepsilon_{\text{ins}}{)}^{\left(\#\;\text{insertions}\right)}\prod_{j\in \text{Matches}(𝒜)}(1-10^{-q(j)∕10})\prod_{j\in \text{Mismatches}(𝒜)}(10^{-q(j)∕10})$$

for any feasible alignment 𝒜. The parameters *ε*_ins_,*ε*_del_ are specific to the sequencing machine and may depend on whether the reads are forward or reverse in the mate pair.

Finally, conditional on ${𝒜}_{t,i}$, each mismatched/inserted base in read *r_t,i_* is sampled uniformly at random; the likelihood of the read’s nucleotides *s_t,i_* is the product

$$p(s_{t,i}\;|\;{𝒜}_{t,i})={\left(\frac{1}{4}\right)}^{\#\;\text{insertions}}{\left(\frac{1}{3}\right)}^{\#\;\text{mismatches}}\prod_{j\in \text{Matches}}1\{s\;\text{and}\;f\;\text{match at}\;j\}.$$

### ChronoStrain’s Database (and a construction for *E. coli*)

In ChronoStrain’s model, a strain s∈𝒮 is simply a (multi-)set of markers ${ℳ}_{s}$, where a “marker” $m\;\in \;{ℳ}_{s}$ is simply a nucleotide sequence specific to that strain. Such a sequence could be, for example, a variant of a known gene encoding a particular function. We pay no attention to their ordering on the chromosome, but we do care about their multiplicity and their exact nucleotide sequence.

A key assumption required of our database (implicitly encoded in Equation 1) is that each ${ℳ}_{s}$ includes all substrings of s’s chromosomes that share sufficiently high % identity with reads. All such sequence fragments must be accounted for, even if these mapped regions do not necessarily correspond to the genes that the seeds were derived from. Therefore, satisfying this constraint requires some careful consideration, especially at the strain level where (for instance) we estimate *E. coli* strain proportions as in our work.

To specify database marker seeds, we require a FASTA record specifying a reference sequence for each seed. For the specific purposes of *E. coli* strain abundance estimation found in this work, our seed of marker genes were derived from the following sources:

1. all genes from MetaPhlAn’s [13] database with *E. coli* clade designation (at the time of this publication, we used v31 of the MetaPhlAn3 database),
2. genes from Institute Pasteur’s MLST scheme,
3. genes used in Clermont’s method of phylogroup classification, and
4. the fimH fimbrial gene, which is a well-studied island of pathogenic mutations. [39]

Assuming each strain contains *O*(1) sequences that closely match each marker seed, the database size grows roughly linearly in the product (# Strains) x (# Marker Seed Length).

For group 1, we used the reference sequence provided by the corresponding MetaPhlAn database. For 2 and 4, the corresponding reference nucleotide sequence for each gene was extracted from NCBI’s genbank annotations of the chromosomal assembly of strain K-12, substr. MG1655. For group 3, for which we started with PCR primers, the extraction was more involved. First, we exhaustively searched for instances of forward/reverse primers on all available *E. coli* strains in NCBI’s RefSeq database, and pulled out each gene’s instance for all strains. Then, for each gene name, a reference was picked by performing a multiple alignment via MAFFT [40], and determining the instance which minimized the aligned Hamming distance to all others. Before proceeding, we removed trpA and trpB from Clermont’s listing (group 3), since they are also provided in group 2.

We noted up to ~80% sequence similarity between these *E. coli* reference seeds and genes within the family level (e.g. *chuA* gene in *Citrobacter* and overall genome-wide similarity to *Shigella*). These must be accounted for to avoid as much model misspecification as possible. To account for within-strain similarities, as well as similarities *outside* the species level, we performed a BLAST query onto a local database of isolated NCBI RefSeq strains from the *Enterobacteriaceae* family. For each gene query, the results were thresholded at 75% identity and minimum length 151 (the median length of reads), and set to report a generous number of hits (--max_target_seqs = 10 times the number of database genomes). Since the genes were queried at the genus level, our database naturally includes strains that are not *E. coli* (e.g. *Shigella, Klebsiella* and *Salmonella*), even if we did not originally set out to resolve these other species. We note that in the analyses found in this work, we purposely do not render abundance trajectories of strains that are not *E. coli*.

To reduce the redundancy of NCBI’s collection of RefSeq strain genomes, we ran a clustering heuristic. First, for each marker gene, we performed a multiple alignment using MAFFT between each of its BLAST hits from all strains; if a strain originally had more than one BLAST hit for a given marker, we used the top reported result. We concatenated all of these multiple alignments, and constructed a matrix of Hamming distances between each pair of strains. Using this as input, we ran scikit-learn’s implementation of agglomerative clustering (with “average” linkage) to generate clusters of strains. The distance threshold was set to 0.3% of the concatenated alignment length, corresponding to 99.7% ANI with respect to the marker sequences. Each cluster 𝒞’s representative strain $s_{\text{rep}}(𝒞)$ was chosen as the strain which most closely resembled the whole cluster, in terms of the Hamming distance to all other clusters. More specifically:

$$s_{\text{rep}}(𝒞)=\underset{s\in 𝒞}{\text{argmin}}\sum_{{𝒞}^{\prime }}|d(𝒞,{𝒞}^{\prime })-d(\{s\},{𝒞}^{\prime })|$$

where $d(𝒞,{𝒞}^{\prime })=\frac{1}{|𝒞||{𝒞}^{\prime }|}\sum_{x\in 𝒞,y\in {𝒞}^{\prime }}d(x,y)$ is the average Hamming distance between two clusters. After this process, we ended up with a database of 1225 *Enterobacteriaceae* strain cluster representatives and their marker sequences. The complete database is input into the model, even if only about half (623) are *E. coli*.

Finally, we performed a pruning step, during which we enumerate all pairs of markers which overlap on the corresponding reference genomes. If such a pair exists, we simply merge them into a new marker. Note that overlaps in markers are a side effect of having to define our markers via sequence identity and not by gene annotation. We do this because during our inference algorithm, reads that map to overlapping regions should only count once in the likelihood function; without any sort of correction, they have the potential to be counted multiple times. After all is said and done, each strain entry in the database covers ~0.4% of a typical *E. coli* chromosome (the median length of the *E. coli* chromosome is around 5 Megabases).

### Read Filtering

The read collection *R* of reads that map to database marker sequences is determined via alignment. Note that this step merely serves as a first-pass filter; exact analysis of these alignments and multiple mapping positions are left for the calculation of model parameters (Supplemental Text §B.1.1). In this step, we only require a one-sided test, in which we rule out a great majority of reads which definitely do not map to some marker of a known strain. Thus, out of speed considerations, we use bowtie2 using the --very-fast, --local setting with a few changes. Alternatively, our software does support bwa [41] and bwa-mem2 [42] as built-in options, but more generally any other alignment program which outputs a standardized SAM format can be substituted here.

We lowered the seed length to *k* = 15 to increase robustness to more densely populated (novel) SNVs or indel errors, without greatly sacrificing runtime. The alignment parameters were chosen specifically to suit our needs. More precisely, the match/mismatch penalties were assigned the log_2_-odds ratio of errors from the PhredWithIndels model (assuming a rather pessimistic quality score of 20) in relation to a uniformly random sequence of nucleotides:

$$\;\text{Match Bonus}=2\approx \log_{2}\left(\frac{1-10^{-2}}{1∕4}\right)\;\text{Mismatch Penalty}=-5\approx \log_{2}\left(\frac{(3∕4)\times 10^{-2}}{1∕4}\right)$$

Assuming that indels are randomly distributed across each read according to indel error rates *ε*_ins_, *ε*_del_ (Supplemental Text §B.2), we set the gap open penalty to zero and the extend penalties to −log_2_(*ε*_ins_), −log_2_(*ε*_del_). Note that these parameters effectively tell the alignment program to solve a certain maximum-likelihood problem, approximately in line with what the method needs for read filtering and model parameter calculation.

Based on these alignments, we only kept reads that aligned to some marker sequence, where the alignment mapped with at least 97.5% identity. The % identity is calculated in relation to the entire read, even if the alignment program used produced local alignments (we extrapolate local alignments into fitting alignments by adding clipped bases back in). We also remark that there was a big overlap between the outputs of bwa mem versus bowtie2, with minimal differences in the output after inference. The advantage of bowtie2 with our chosen settings is that it is an order of magnitude faster, particularly for larger datasets (≥ 20 million reads per timepoint).

### Target Posterior Approximation

Based on the Bayesian generative model, we aim to estimate the posterior *p*(*X* ∣ *R*). We approximate this distribution using a PyTorch implementation of ADVI [26], which uses stochastic optimization on Monte-Carlo estimates of the evidence lower bound (ELBO) objective. By default, we eschew a mean-field factorization. Instead, the implementation optimizes a reparametrized fully-joint (∣𝒯∣∣𝒮∣)-dimensional Gaussian approximation (a “fully non-diagonal” covariance matrix); this is because we specifically wanted as much time-series coherence as possible in the posterior samples. The disadvantage is that it incurs a slight extra cost in memory, although we find that we were still well within the practical range of a modern personal computer (See Methods - Comp. Resources). For the scenario where the user only needs *marginal* statistics which don’t require time-series correlation in the samples, we implemented partially mean-factorized posteriors as options to be provided in the software; these have a much smaller memory footprint (O(∣𝒮∣) or O(∣𝒯∣) depending on the type of factorization). Regardless of the choice of factorization, in order to make this algorithm scale with database size, we implemented an approximation strategy which utilizes sparsification of the model (outlined in Supplemental Text §B).

The objective function was optimized using PyTorch’s implementation of Adam, which enables us to accelerate the inference on a GPU. Our strategy relies heavily on sparse matrix calculations. The default settings, equivalent to the settings that we used for this paper, are as follows. The posterior approximation of *X* is initialized to have mean zero (corresponding to a uniform abundance profile for all timepoints) and covariance equal to the identity matrix. The learning rate decay schedule *η*(*t*) was initialized to *η*(0) = 10^−3^. This was set to decay by a factor of 0.25 when the ELBO value no longer improved between epochs (1 epoch = 50 iterations) after at least ten rounds. The optimization was set to stop when *η* was less than 10^−5^. These settings were decided based on empirical observations, or more precisely on whether or not the gradient optimizer reliably improved the ELBO throughout the run. If for a particular dataset the ELBO trends downward at any point, we intend for the user to manually decrease the learning rate.

### Fully Synthetic Benchmark

The fully synthetic benchmark was designed to demonstrate that ChronoStrain can potentially attain high sensitivity in the low-coverage regime, even when the relevant strains are only a few SNVs apart. The primary assumption for this experiment is that the single-nucleotide differences are contained within the database marker sequences; thus closing the gap between what is possible using markers and the entire (pre-assembled) chromosome.

To generate the artificial variant, we edited positions 4533232-4533239 (middle of an annotated fimbrial gene) of the reference chromosome into a string of T’s. Three of these nucleotides were already T’s in the reference, so this results in only five altered nucleotides that are more or less contiguous.

We simulated Illumina reads using Art [43] from two strain genomes: a RefSeq chromosome (BW25113), and an edited version with 5 edited loci on the fimbrial gene (Figure 2a). To introduce noise, we used the software authors’ provided HiSeq error profile and randomly chose the read counts per strain for each timepoint *t* to be Multinomial(*N*, ${\vec{y}}_{t}$)-distributed, where ${\vec{y}}_{t}\;=\;(y_{t,1},y_{t,2})$ is the relative abundance profile of the simulated strains at time *t*. The specific choice of reference genome and the fim gene is arbitrary and is not expected to impact the results, because the genomes of the two variants are meant to be completely identical outside of the edited loci.

Each method was provided a database consisting of *only* the two simulated variants. Due to the amount of similarity in the fully synthetic benchmark, we gave the comparator methods some advantages by using non-default settings. First, when running the strain detection component (StrainGST) of StrainGE for the synthetic experiment, we needed to disable the minhash compression heuristic via the --fulldb option. This was done because the *k*-mer profiles of the two strains were identical after compression; this is a lossy step and is not meant to be used in cases of extremely high genetic similarity such as our synthetic setup. Second, we observed that StrainEst tossed out all five of the relevant genomic loci from the synthetic dataset due to a lack of coverage, and thus we disabled the thresholding option.

In Figure 2c, the error is computed by treating each relative abundance profile as a probability vector over strains and computing the total variation distance to the ground truth per sample (0.5 times the *ℓ*_1_-distance). This constrains each sample’s error between 0 and 1.

### Semi-synthetic benchmark

For our semi-synthetic experiment, we simulated the reads using the same scheme as in the synthetic datasets. We made an effort to construct a customized database for each comparator method, so that each represents the exact same collection of reference strains (keeping in mind that some databases store more information about these strains than others). StrainEst’s databases are meant to be specific at the species level, and thus we first removed all non-*E. coli* strains from ChronoStrain’s clustered database (Methods - Chronostrain’s Database). Using these strains’ RefSeq identifiers, we retrieved the whole assembled chromosome and constructed fresh databases for StrainGE and StrainEst. For both types of databases, we skipped their respective database clustering, since we had already performed a clustering beforehand. Note that in StrainEst’s original paper [27], the authors recommend a far coarser grouping to improve runtime. However, we found that even if we performed an *additional* coarsening on top of what was already done, it only provided an improvement of 15-30 minutes per sample. All of the above was done by using a 99% nucleotide identity agglomeration of ChronoStrain’s database. The more fine-grained agglomeration of 99.7% identity used for analyzing the UMB dataset roughly doubles the number of *E. coli* representatives, which made StrainEst costly to run. The 99.7% similarity database results in ChronoStrain’s database size and inference runtimes roughly doubling (but still within competitive range), whereas StrainGST’s runtimes stays more or less constant.

To maintain what is likely to be the way users will run these methods on real data, we tried to stay as close as possible to the default, manuscript-specified pipeline and settings for each method. A minor exception was made for StrainGST: by default, the program detects at most five strains; we raised this value to six (the four simulated strains, plus the number of strains output from the real data alone).

For measuring the error in abundance estimation, we again computed the total variation distance after re-normalizing the abundances estimated for the four in-silico reference genomes. To derive a metric for the *classification* part of the task, we chose the recall rate which measures the fraction of (timepoint *t*, strain ID *i*) pairs for which each algorithm detected strain *i* at timepoint *t*. StrainEst and StrainGST both report zeroes for strain IDs, hence “detection” can be defined as strain *i* having a nonzero abundance estimate, while for ChronoStrain we used the criterion described in Methods - Detection Classifier.

### Detection Classifier

ChronoStrain’s posterior trajectories do not include any zeroes due to use of the softmax transformation; we have not yet introduced any sort of zero-inflation component to our model (but abundance values can be arbitrarily close to zero, e.g. 10^−50^). For the purposes of comparison to the other methods, we introduce a general definition: say that a strain is detected at the “(*q*, *τ*)-threshold” if the posterior’s *q*-quantile is above a threshold *τ*. In this work, we chose *q* = 0.025 and τ=1∕∣𝒮∣, so that “detection” means that the centered 95% credibility interval (CI) of the abundance of *i* is strictly above the uniform abundance profile, which is the initialization point of the variational optimization.

This was chosen as a reasonable tradeoff between sensitivity and specificity for displaying “binary” classifications, such as in Figure 4. Note that this provides a reasonably high sensitivity only if the database has many strains (e.g. ∣𝒮∣ is large). Simultaneously, the requirement that the *entire* 95% CI be above this threshold is very stringent and may lead to false negatives, especially when there is ambiguity between similar taxa (e.g. participant UMB19 in Supplemental Figures). In general, the raw posterior distribution output by ChronoStrain are more useful than any derived binary classifier; the latter should be used for high-level summarization.

### Computational Resources

For benchmarking, all three methods were run on identical, high-end hardware (12900KF with 128 GB of DDR5 memory). ChronoStrain, in particular, is designed to be run on a GPU with minimal CPU footprint (can optionally be run on the CPU itself with 10x~20x slowdown). Other benchmarked methods were not designed with GPU hardware in mind. For our experiments, the CPU footprint (with GPU acceleration) was up to 2.5 GB. Inference was boosted by a single RTX 3090, which contains 24 GB of graphic memory. This was the primary limiting resource for the full UMB dataset (up to ~22 GB peak usage), but we saw that each semisynthetic run (five timepoints from a UMB participant) only used up to ~6 GB. Our software has a future planned update to enable distributed computing across multiple GPUs, thus enabling datasets with more input reads and a bigger database.

### Sequencing & Real Data Processing

MacConkey-cultured samples were sequenced in the same manner outlined for the original UMB dataset [7]. Starting with the raw reads, we used the demultiplexed, whole-genome portion of the UMB dataset for all participants. Read pre-processing was done via the KneadData pipeline (v0.11.0, https://huttenhower.sph.harvard.edu/kneaddata/) to trim adapters and low-quality bases at the ends (phred ≤ 10) via Trimmomatic [44] and discard reads which align to the human genome via Bowtie2 [28].

## Supplementary Material

Supplement 1

Supplement 2

## Footnotes

1 We did not run StrainEst because its runtime made a full-scale analysis infeasible. Its whole-genome alignment approach introduced issues that we were unable to correct for; see Supplemental Text §A.1 for a discussion.
