# Supplementary material for: Strain Tracking with Uncertainty Quantification
Source: bioRxiv. 2023 Jan 26:2023.01.25.525531. Preprint. [Version 1] doi: 10.1101/2023.01.25.525531 (PMC9900846; doi:10.1101/2023.01.25.525531)
Supplement: Supplementary file 1 [file media-1.pdf]

Supplemental Text

A Related Works

According to the overview of strain detection methods in [1], ChronoStrain can be categorized as a *read alignment-based* method. Here, we focus on the *abundance estimation* aspect of strain inference (which is different from *detection*), and thus organize the methods slightly differently. More specifically, we emphasize three orthogonal features of existing methods: (1) *whole-genome based* versus *gene based*, (2) *read modeling* versus *allele frequency modeling*, and (3) *genotype-learning* versus *reference calling*.

In this work, we largely omit from the discussion methods which rely on metagenomic assembly. This is because assembling *low-abundance* strains (such as UPEC in the gut) requires an extremely high read depth to be successful. Furthermore, existing methods [2] that nevertheless attempt this strategy produce contigs that are binned into metagenome-assembled genomes (MAGs). However, when genomes exhibit sufficient *overall* similarity — such as with phylogroup B2/F/G *E. coli* strains — it can be challenging to resolve contigs into MAGs correctly. Still, the general idea of using assembly (or a related algorithm) using reads still has some merit; we mention it in Section A.3.

A.1 Whole Genome vs Gene-based methods

Methods that use whole genome information sometimes rely on genome-to-genome alignments; examples include StrainEst [3], which relies on pairwise alignments, and the marker gene database construction step in BIB [4] (discussed below). In general, there is a limitation that applies to *any* method that relies on a genome-to-genome alignment, because they can be confounded by large-scale rearrangements and recombinations, even across variants of the same species. Indeed, these events are not uncommon in *E. coli*, a major focus of this paper. On the other hand, some multiple alignment algorithms that try to account for this issue (such as progressiveMauve [5]) simply fail to scale with input size; the problem is far from being resolved at this point in time. Two examples of whole-genome based methods that do not use genome (pairwise or multiple) alignments are Sigma [6] (which only uses read-to-genome alignments) and StrainGE [7] (which uses k-mer decompositions).

Another approach that avoids this issue altogether is to associate taxonomies with a single gene (e.g. 16S for Karp [8]), or collections of genes (e.g. ConStrains [9], StrainFinder [10], the inference algorithm in BIB [4] and our method ChronoStrain). Beyond using just the 16S gene, the task of finding useful collections of genes is implemented by MetaPhlAn [11], which defines a group of "core" genes for each clade. Either approach enables gene-specific variant calling to define strains (StrainPhlAn [12], which produces pile-ups and does not resolve strain genotype-specific abundances; see the discussion below about allele frequency-based methods).

Our method is built on the latter idea, with one key difference: our belief is that the genes encoding interesting phenotypic signatures (e.g. hypothesized pathogenicity of UPEC strains) are precisely those that are *not* core to the species. In fact, this is the same reason why whole-genome methods have been developed; but our approach fits nicely in between the two extremes and has the potential to utilize a larger (or more clinically relevant) fraction of the pan-genome.

A.2 Read Modeling vs Allele Frequency Modeling

The phrase *read modeling* refers to methods that directly utilize the read nucleotides, usually in a probabilistic model without any intermediate "proxies" for reads (e.g. BIB, Sigma, Karp and

ChronoStrain). This is in contrast to *allele frequency modeling*, whereby a method will typically align reads to a collection of reference genomes, and attempt to de-convolve a SNV frequency matrix into abundance ratios (StrainFacts, StrainFinder, StrainEst fall into this category).

The primary issue with operating only on allele frequencies is that the action of computing a frequency matrix causes the loss of evidence of SNV-to-SNV correlations that each read provides. Deconvolving frequency matrices into strain genome & abundance profiles is, generally speaking, statistically challenging with just reference information. A useful example to consider is the task of deconvolving a frequency matrix on L loci produced by two distinct genomes at equal abundances; this special setting requires picking one of 2^{L-1} possible combinations of SNVs. Mathematically, this is equivalent to the *haplotype phasing problem* from the context of human genomes [13], for which reads are known to be extremely helpful, and population-wide correlations (e.g. linkage disequilibrium) alone generally cannot solve every instance. Furthermore, when calling alleles across whole genomes (and not gene-specific alleles), one is effectively performing genome-to-genome comparisons, and thus this approach is subject to the same challenges discussed in the previous section.

A.3 Genotype Learning vs Reference Calling

We use the phrase "reference calling" to describe methods that use a reference database of assemblies, and attempt to output abundance estimates of *reference labels* that are most appropriate for the latent strains in the sample. Several methods (e.g. StrainGST, StrainEst, Sigma, BIB and Karp), including ChronoStrain fits in this category. The main limitation with this approach is that it is not obvious what to do for experimental data when there are *novel* strains. (The following points are discussed in the main text, but we recapitulate them here.) It is possible that a new strain may not deserve any *one particular* reference label; it may be mixtures of different known variants across loci, or it may contain previously unobserved variants. Typically, one digs further using allele frequency estimators such as StrainPhlAn and StrainGR (which is packaged with StrainGST), bringing us back to the allele frequency modeling setting. Another alternative is to output mixtures of labels through a posterior distribution (BIB and ChronoStrain); point estimates are also possible (StrainGST, Sigma, Karp), but when comparing across multiple samples, one may end up with inconsistency in the labels.

One potential advantage of several "genotype-learning" methods (ConStrains, StrainFinder, StrainFacts, which all happen to operate on allele frequencies) is that these methods attempt to jointly estimate strain-specific genotypes and abundance ratios. The word "potential" is used to emphasize the fact that this is a *much* more general task than what the reference-calling methods are attempting; thus, the inherent *dimensionality* of these problems is much higher, and there are more ways that these methods can go wrong. It is important to note that this harder problem is not limited to the allele-frequency modeling approach; by utilizing reads directly, one can treat this as a variant of an assembly problem (either *de novo* metagenomic assembly or as an analogue of a *polyploid* haplotype assembly).

ChronoStrain fits squarely into the category of reference calling, which is why it currently outputs mixtures of references on the UMB dataset. We do not attempt to learn genotypes *de novo* in this work, but we leave it as a crucial future direction as discussed in the Discussion section of the main text. To our knowledge, a bona-fide "time-series"-aware (genome or genotype) assembly algorithm across multiple metagenomic samples (e.g. beyond merging samples into one giant read set) has not yet been invented, but we imagine it would be a quite helpful contribution to the field.

A.4 Method-specific Comparison

We note that out of all works mentioned, ChronoStrain is the only one to explicitly encode timepoints t at which samples were taken, even if several other works perform joint estimation across multiple samples (ConStrains, StrainFinder, StrainFacts). Excluding this feature, exactly two methods agree with ChronoStrain for all categories: BIB and Karp (as well as Metakallisto [14] which inspired the latter). We summarize their differences at a high level.

BIB leverages read alignments to a database of core genes (genes shared by all strains of a species or higher clade) via a likelihood model on the read's nucleotides and phred scores; it is the *only* other method known to us that implements an approximate posterior probability distribution over abundance profiles. BIB has two limitations that do not extend to our method. First, its database construction of core genes is seeded by a whole-genome multiple sequence alignment (MSA) and thus has trouble scaling to species with more than a few dozen strains. Second, our method includes a position-encoding, fragment sliding-window model Eq. (1), whereas BIB does not. It was excluded in that method for the sake of computational complexity (since the method wants to fully consider reads that don't map uniquely to their database), but finding a tractable approximation (Section B.1.2) is a key part of ChronoStrain's implementation.

Karp was designed for 16S reads and builds upon Metakallisto's usage of k-mer based pseudoalignments: it was developed after observing that utilizing base quality can be helpful (ChronoStrain also includes quality information). However, Karp's model does not distinguish the scenario when a read maps to a reference in multiple positions; the inherent assumption is that reads map to a unique position for each reference. This assumption is valid if each reference sequence is a single 16S gene variant, but the 16S gene is known to vary in copy number [15] and thus does not result in a neat, one-to-one correspondence with actual genomes. Still, it is not unreasonable to at least try to run this method given the findings of the authors of Metakallisto, which produced great results on synthetic, whole-genome data. However, we found that when Karp was run on real data (UMB18), it showed an incoherent and uninterpretable output across time, and deviated greatly from the two methods discussed in our paper.

In the main text, we draw a comparison between our results and those of StrainGST (an algorithm in the StrainGE package). That method follows a common paradigm in bioinformatics: designing *fast*, low-memory footprint algorithms by working in k-mer space. It operates iteratively, by repeatedly calling successive reference labels whose k-mer profile has the highest "overlap" with a remaining set of k-mers formed by the reads. However, like allele frequencies, decomposing read collections to k-mer frequencies loses *some* SNV-to-SNV correlations, and there is a natural tradeoff. If k is too large, then the speed/memory advantages becomes negligible and the algorithm loses robustness to sequencing noise. If k is too small, one sacrifices some ability to resolve long-range, multi-locus correlations, especially for strains at perpetual low-abundance. As a consequence, untangling the sequences and abundances of multiple similar, co-abundant strains becomes challenging.

In theory, the following latent information ought to be inferrable from raw data, but becomes extremely challenging if one uses algorithms that are lossy, which is the case with allele or k-mer counts. These are: (1) correlation in time-series (*which SNVs are correlated across time?*), and (2) correlation between loci (*which SNVs are correlated across reads?*). It is an algorithmic challenge to efficiently resolve both simultaneously, particularly for low-abundance strains, without resorting to sequencing depths likely far beyond what is theoretically required. This is precisely the type of scenario where Bayesian methods — such as ChronoStrain — tend to shine, and these concerns helped shape our algorithmic design.

A.5 Methods Excluded from Analysis

We remark upon tools mentioned above that did not make it into our benchmarks (fully synthetic and semi-synthetic). First, BIB's database construction did not scale well to the hundreds of genomes being used in the semisynthetic scenario. In contrast, since ChronoStrain's database requires one to specify marker seeds without worrying about copy number or homologies, it took ∼2 hours to construct the full database of ∼5400 *Enterobacteriaceae* genomes and $\frac{1}{2}$ hour to agglomerate this into the final 1225 at 99.7% marker nucleotide identity. Furthermore, the original StrainEst paper ran ConStrains [9] and Sigma [6] for its own comparisons. Unfortunately, in that work, the authors found that these two tools fail to properly learn sub-species mixtures of *E.coli* and/or relies on very high coverages, and thus we do not expect them to perform better than what StrainEst can already accomplish. Furthermore, we could not run ConStrains to completion due to its reliance on outdated software.

We also made an attempt to run at least one estimation tool that provided genotype deconvolutions jointly with abundance estimates. In particular, we ran StrainFacts [16], which is the newest published allele-based method, using the pipeline documented in the manuscript. We could not include it into the fully synthetic benchmark due to a software limitation that the database contains at least five reference strains; that scenario only has two. For the semisynthetic benchmark, we used our collection of ≥ 300 *E. coli* strains from the 99% identity agglomeration (∼650 genomes), and was able to get it to run on the dataset. This came with a caveat: the main StrainFacts algorithm (which outputs a maximum a posteriori estimator) requires specifying a value k which represents the desired number of genotypes. Since we actually mixed the synthetic reads with real reads, this value could not be determined.

Instead, we attempted to give it an advantage by passing in **only** the simulated reads, with $k = 4$ passed as input. Note that the method attempts to output abundance estimates of potentially *novel* combinations of alleles, which has no guarantee of perfectly matching our ground truth strains. To engineer a fair metric for this purpose, we calculated the following, adjusted ℓ^1 error metric

$$\text{Error}(\hat{x}) = \min_{\pi \in S_4} \sum_{t \in \mathcal{T}} \sum_{i=1}^{4} |x_t(i) - x_t(\pi(i))|$$

where S_4 is the set of all permutations of four elements, x is the ground truth abundance ratio, and $\hat{x}$ is the abundance estimate. This is the same error metric applied to the other methods, but minimized across all ways to mix-and-match the inferred genotypes with the ground truth strains. Unfortunately, it consistently provided poor results (errors between 4 and 5 across all coverages, indicating that the algorithm did not actually learn the simulated strains) for all coverages.

This suggests a potential incompatibility or limitation of allele deconvolution methods when using the simulated benchmarks. For instance, it is quite possible that StrainFacts learned at least one "fuzzy" genotype (mixtures of alleles per loci) that was a mixture of the simulated strains. This is problematic, since it is rather unclear how the four fuzzy genotypes relate to the four synthetic genotypes, and since it is not clear how to further untangle these into individual abundances. (Our main hope was that the cross-sample correlation provided sufficient information to resolve non-fuzzy genotypes from the allele frequency matrix.) Another potential issue might have been that the software was designed using a bi-allelic assumption, whereas the true synthetic genotypes — and the underlying database — are multi-allelic after multiple genome alignment. Keeping these issues in mind, we stopped short of re-engineering parts of the code ourselves. The core problem of allele deconvolution is a strictly harder one than what we are after (after incurring some information loss by converting reads into allele

counts, as discussed in Section A), so our benchmarks' assumptions/requirements may have been too optimistic.

B Mathematical Details

B.1 Objective Function

The posterior that we are after is $P(X \mid R)$ (conditional on R aligning to the database), where R is the subcollection of reads for which we condition on as originating from our markers. As mentioned in the main text, our algorithm is an adaptation of Automatic Differentiation Variational Inference (ADVI) [17]. A core ingredient of this method is the Monte-Carlo estimate to the Evidence Lower Bound objective (ELBO):

$$\begin{aligned} \mathsf{ELBO}(\varphi) &= \mathbb{E}_{X \sim q_\theta}[\log p(R, X)] + H[q_\varphi] \\ \rightarrow \widehat{\mathsf{ELBO}}(\varphi) &\approx \left(\frac{1}{M} \sum_{m=1}^{M} \log p\left(R, \widetilde{X}^{(m)}\right) \right) + H[q_\varphi] \end{aligned}$$

where q_φ is an approximating likelihood function parametrized by φ, $\widetilde{X}^{(1)}, \dots, \widetilde{X}^{(M)}$ are i.i.d. samples from q_φ, and $H(\cdot)$ is the Shannon entropy function (explicitly computable for Gaussians). Note that the above is not "stochastic optimization" in the commonly understood sense of Machine Learning literature [18], since we do not sub-sample the reads for optimization (we do a full pass on the entire dataset). Subsampling would help scale the optimization to much larger datasets, but the above calculation helps minimize variability across optimization seeds.

The core principle of ADVI is that one maximizes this function by pushing it through standard, automatic-differentiation algorithms as a black box. Since such a heuristic would call for evaluating this function many times — preferably using fresh samples for each iteration — it is critical to minimize the computational complexity required to estimate the ELBO. Otherwise, this becomes a rather expensive algorithm which might not run, even on high-performance computing resources. This is in terms of runtime *and* in terms of the memory required when storing all of the necessary gradients.

We assume that the likelihood of a read set R given latent X can be written as the product

$$\begin{aligned} p(R \mid X) &= \prod_{t \in \mathcal{T}} p(R_t \mid X_t) && \text{(Cond. Indep. over } \mathcal{T}\text{)} \\ &= \prod_{t} \prod_{i=1}^{N_t} p(r_{t,i} \mid X_t) && \text{(Cond. Indep. over reads).} \end{aligned}$$

Each individual term can be expressed as a marginalization over fragments, e.g.

$$p(r_{t,i} \mid X_t) = \sum_{f_{t,i}} p(r_{t,i} \mid f_{t,i}) p(f_{t,i} \mid X_t).$$

Dropping the subscripts (t, i) for readability, we expand this according to the model described in Methods:

$$\begin{aligned} p(r \mid X_t) &= \sum_f p(r \mid f) \sum_\ell p(f \mid Y_t, \ell) p(\ell) \\ &= \sum_f p(r \mid f) \sum_\ell p(\ell) \left(\frac{\sum_{s \in \mathcal{S}} Y_t(s) n_{f,s}^{(\ell)}}{\sum_{\hat{s} \in \mathcal{S}} Y_t(\hat{s}) n_{\hat{s}}^{(\ell)}} \right) \\ &= \sum_{s \in \mathcal{S}} Y_t(s) \sum_f p(r \mid f) \left(\sum_\ell p(\ell) \frac{n_{f,s}^{(\ell)}}{\sum_{\hat{s} \in \mathcal{S}} Y_t(\hat{s}) n_{\hat{s}}^{(\ell)}} \right) \end{aligned}$$

This expression for $p(r \mid X_t)$ looks complicated, but it can be broken down into two major pieces.

1. The calculation of fragment-to-error likelihoods $\varepsilon_{r,f} \overset{\text{def}}{=} p(r \mid f)$, and
2. The calculation of the (weighted) strain-specific fragment frequencies

$$\omega_{f,s} \overset{\text{def}}{=} \sum_{\ell} p(\ell) n_{f,s}^{(\ell)} \left(\frac{1}{\sum_{\hat{s} \in \mathcal{S}} Y_t(\hat{s}) n_{\hat{s}}^{(\ell)}} \right)$$

In plain English, the first piece characterizes the *error likelihood of reads* enumerated across the reference strains, and the second piece characterizes the *genetic diversity* within and across reference strains. The inverse term (containing the sum indexed by $\hat{s}$) can be understood as a correction that accounts for the bias induced by the choice of marker seeds. Roughly speaking, when estimating the posterior distribution, this term ensures that strains aren't unfairly boosted just by the pure virtue of having more marker sequences.

Let $\mathcal{F}$ be the collection of all possible fragments in the model. Algorithmically, if we are given both pieces in the form of two matrices

$$W = \left(\omega_{f,s} \right)_{f \in \mathcal{F}, s \in \mathcal{S}} \qquad \text{and} \qquad E_t = \left(\varepsilon_{r,f} \right)_{r \in R_t, f \in \mathcal{F}}$$

then the likelihood computation can be reduced to the evaluation of the product $E_t^\top W Y_t$ across $t \in \mathcal{T}$. Symbolically, this is a simple linear algebraic operation, and thus easily black-boxed using standard tensor libraries.

In practice, however, the matrices W and E can be extremely large. Furthermore, E can be pre-computed but W cannot, since the latter explicitly depends on the Y_t's (for which we will use samples throughout ADVI). The key observation is that for biologically plausible models (e.g. variants of markers need to be somewhat similar), W can be approximately decoupled from Y_t via a sparse sum and E_t is "close" to being sparse, in the sense that all but $O(|R_t|)$ entries are vanishingly close to zero. We carefully designed heuristics for sparsely approximating E_t (Section B.1.1) as well as W (Section B.1.2), which are key ingredients for scaling up our model to accommodate the entire *E. coli* reference database.

B.1.1 Per-read error likelihood calculation

In this section, we explain how we estimated the matrix E_t. The overall goal here is to identify which rows f support the row vector corresponding to read $r_{t,i}$. For the sake of exposition, we drop the subscripts and write $r = (\sigma, q)$ to represent a generic read, where σ is the read's nucleotide sequence. Expand

$$\begin{aligned} \varepsilon_{r,f} &= p(r \mid f) \\ &= \sum_{\text{Alignments } \mathcal{A}} p(\sigma \mid f, \mathcal{A}) \times p(\mathcal{A} = a \mid f; q) \end{aligned} \tag{S.1}$$

for the error likelihood, which includes marginalization over $\mathcal{A}$. Instead of exhaustively listing out all theoretically plausible f, we narrowed down the search using an alignment-based heuristic.

The main idea here is that we can restrict the calculation to candidate fragments f and alignments a for which this likelihood value is significantly larger than their alternatives. More precisely, this heuristic assumes the right hand side of Equation S.1 is dominated (by several orders of magnitude) by a single term:

$$\varepsilon_{r,f} \approx p(\sigma \mid f, \mathcal{A}^*) \times p(\mathcal{A}^* \mid f; q)$$

where $\mathcal{A}^*$ is an optimal global alignment between f and σ. For those reads r for which all of the above products are sufficiently small ($< e^{-500}$), we simply round $\varepsilon_{r,f}$ to zero.

Note that this truncation applies on a *per-fragment* basis for each read, so we must search for all candidate fragments f which align well to r. To carry out this strategy, we aligned the filtered reads to the marker database. Since speed is less of a concern than in the filtering step operating on *all* of the reads, by default we use *bwa mem* (and optionally *bwa-mem2*) which has been suggested for providing better alignments when genomes in the database are similar to each other [19]. We use the same parametrization as in the filtering step – but this time configured to output all available alignments instead of just the best one. Each alignment ought to be understood as a mapping to a particular marker m in the database. If the alignment clipped read nucleotides that extended inside the boundary of m, we re-included those bases into the alignment without indels; this is to ensure that we properly model the *whole* read. The aligned marker's substring, with gaps removed, was included in the model as a candidate fragment f (the support of r).

B.1.2 Fragment frequency

In this section, we describe how we compute the matrix W, whose entries are $\omega_{f,s}$. Here, s denotes an arbitrary strain, but we may now assume that f is a supporting fragment of E_t for some $t \in \mathcal{T}$. We rewrite each entry $\omega_{f,s}$ for convenience:

$$\omega_{f,s} \overset{\text{def}}{=} \sum_{\ell=0}^{\infty} p(\ell) n_{f,s}^{(\ell)} \left(\frac{1}{\sum_{\hat{s}\in\mathcal{S}} Y_t(\hat{s}) n_{\hat{s}}^{(\ell)}} \right).$$

Note that $n_{f,s}^{(\ell)} = 0$ if $\ell < |f|$, since each fragment f can only be induced by windows at least as long as f. There is some room for truncation in the above sum. Since the negative binomial distribution (using the parameters that we set) is approximately Gaussian, taking ℓ ranging from 0 to $\mu + 2\sigma$ (in reality $|f|$ to $\mu + 2\sigma$), already captures roughly 98% of ℓ's probability mass.

To evaluate $n_{f,s}^{(\ell)}$, we ran `bwa fastmap` to search for exact matches of f within the database. Since the only way f can be induced by a window longer than $|f|$ (by removing padded bases) is if it maps to an edge on a marker, we take

$$n_{f,s}^{(\ell)} = \sum_{m\in\mathcal{M}_s} \sum_{h\in H_f} \mathbb{1}\{h \text{ maps } f \text{ to the edge of } m\} \vee \mathbb{1}\{|f| = \ell\}$$

where, as a reminder, $\mathcal{M}_s$ is the set of markers in strain s, H_f is the set of exact-match mappings to the marker sequences, and $\vee$ denotes the binary OR operator.

Next, we consider the denominator. Note that we can expand the expression by explicitly computing $n_{\hat{s}}^{(\ell)}$:

$$\begin{aligned}\sum_{\hat{s}\in\mathcal{S}} Y_t(\hat{s}) n_{\hat{s}}^{(\ell)} &= \sum_{\hat{s}\in\mathcal{S}} Y_t(\hat{s}) \sum_{m\in\mathcal{M}_s} (|m| + \ell - 2\beta + 1) \\ &= \left(\sum_{\hat{s}\in\mathcal{S}} Y_t(\hat{s}) L_s \right) + (\ell - 2\beta + 1) \left(\sum_{\hat{s}\in\mathcal{S}} Y_t(\hat{s}) |\mathcal{M}_s| \right)\end{aligned}$$

These terms have simple interpretations. The first sum is the (weighted) mean total marker content across strains in the database. The second term is the total number of padded window positions that we introduce; in particular, β is the budget parameter that determines how willing we are to incorporate edge-mapped reads into our model.

Since with high probability ℓ cannot be too large (e.g. consider the rationale behind the suggested truncation $\ell \leq \mu + 2\sigma$), and assuming that the total *length* of markers for each strain typically greatly exceeds the total number of padded bases (which is generally true in our case), we provide the approximation

$$\frac{1}{\sum_{\hat{s}\in\mathcal{S}} Y_t(\hat{s}) n_{\hat{s}}^{(\ell)}} \approx \frac{1}{\sum_{\hat{s}\in\mathcal{S}} Y_t(\hat{s}) L_s}$$

In particular, this expression does not depend on ℓ, and thus we end up with

$$\omega_{f,s} \approx \left(\frac{1}{\sum_{\hat{s}\in\mathcal{S}} Y_t(\hat{s}) L_s}\right) \sum_{\ell=|f|}^{\mu+2\sigma} p(\ell) n_{f,s}^{(\ell)}$$

In log-likelihood space (which we need for numerical stability when computing the ELBO), the expression is

$$\log \omega_{f,s} \approx \log\left(\sum_{\ell=|f|}^{\mu+2\sigma} p(\ell) n_{f,s}^{(\ell)}\right) - \log\left(\sum_{\hat{s}\in\mathcal{S}} Y_t(\hat{s}) L_s\right)$$

We gain quite a bit from this chain of heuristic reasoning. The primary advantage here is that the above expression is now *decoupled*:

- the complicated summation over ℓ does not depend on Y_t, so it can be *precomputed*, and
- the second term is easily computable via a single vectorized operation during ADVI.

Furthermore, from a purely algorithmic perspective, this expression is rather nicely interpretable. The first term can be thought of as the "bag-of-words" component from a standard topic model ($\log \mathbb{P}(\text{word} = f \mid \text{topic} = s)$). The second term corrects for database-specific bias; it penalizes strains that are over-represented in terms of marker content. Indeed, having too few markers is *not* an intrinsic property of the strain, it is a property of the database being used.

B.2 Model hyperparameters

The negative binomial parameters r, p for fragment lengths were fit using a standard regression package from `statsmodels`. As a proxy to the "true" fragments, we fit parameters to the empirical distribution of read lengths of five arbitrarily chosen participants (the first five test set participants in the cohort), after trimming the adapter sequences. The resulting fitted Negative Binomial distribution has a mean $\mu = 150.5$ and standard deviation $\sigma = 12.27$. This overestimates the variance of read lengths – but keep in mind that a read model is not what we are after. We are actually trying to parametrize *fragment* length (the reference window that the read is measuring). Since edge effects and indels cause variation in fragment lengths in the model, we took this over-dispersed fit to be rather desirable. The window overlap ratio b that parametrizes $\textsc{WindowsWithPaddings}$ is chosen to be 0.5. In words, this means that for each fragment-read pair (r, f) allowed by the model, the fragment f aligns with at least half of the read.

The insertion and deletion error rates $\varepsilon_{\text{ins}}, \varepsilon_{\text{del}}$, depending on whether the read was forward or reverse in the pair, are set by default to the empirical insertion and deletion error rates (on the order of 10^{-6}) from [20]. We remark that that work used a different HiSeq dataset to obtain estimates. However, we do not expect the inference results of this work to be too sensitive to this choice using the given *Escherichia* database, in the sense that changing this setting within an order of magnitude empirically does not alter the results too much. This is something we also observe in practice.

B.3 An estimator for overall relative abundance

Our method is applied to the UMB dataset specifically to estimate the ratio of strains $Y_t = \texttt{softmax}(X_t)$ with respect to only the database strains. Since the whole population of strains, including those species not in the database, is unknown, we derive a simple estimator for the overall relative abundance (e.g. with respect to the entire sample) via the calculation

$$\begin{aligned}\mathbb{P}(\text{Strain} = i \mid X_t) &= \mathbb{P}(\text{Strain} \in \textit{DB} \mid X_t) \times \mathbb{P}(\text{Strain} = i \mid \text{Strain} \in \textit{DB}, X_t) \\ &\approx \left(\frac{n_{\text{DB}}}{n_{\text{marker}}}\right) \times \left(\frac{n_{\text{marker}}}{n_{\text{total}}}\right) \times (Y_t)_i.\end{aligned}$$

Here, given a particular timepoint t, n_{DB} is the number of reads that map to all database chromosomes, n_{marker} is the number of reads that map to markers, and n_{total} is the total number of reads. This formula considers the probability that a randomly chosen strain from the (unknown) overall population at time t is equal to i, an arbitrary *Escherichia* strain.

The goal is to estimate the above *without* performing yet another costly alignment of all reads to the reference collection (e.g. for estimating n_{DB}). We estimate the first ratio as the ratio of overall sequence material available in the population:

$$\left(\frac{n_{\text{DB}}}{n_{\text{marker}}}\right) \approx \frac{\text{Total length of DB genomes}}{\text{Total length of DB markers}} = \frac{\sum_j (Y_t)_j \times (\text{Len of } j\text{'s chromosome})}{\sum_{j'} (Y_t)_{j'} \times (\text{Total len of } j'\text{'s markers})}$$

For the second ratio, we simply count the number of marker-aligning reads and the overall read depth.

Supplement References

1. Anyansi, C., Straub, T. J., Manson, A. L., Earl, A. M. & Abeel, T. Computational methods for strain-level microbial detection in colony and metagenome sequencing data. *Frontiers in Microbiology* **11,** 1925 (2020).
2. Quince, C. *et al.* DESMAN: a new tool for de novo extraction of strains from metagenomes. *Genome biology* **18,** 181 (2017).
3. Albanese, D. & Donati, C. Strain profiling and epidemiology of bacterial species from metagenomic sequencing. *Nature communications* **8,** 1–14 (2017).
4. Sankar, A. *et al.* Bayesian identification of bacterial strains from sequencing data. *Microbial genomics* **2** (2016).
5. Darling, A. E., Mau, B. & Perna, N. T. progressiveMauve: multiple genome alignment with gene gain, loss and rearrangement. *PloS one* **5,** e11147 (2010).
6. Ahn, T.-H., Chai, J. & Pan, C. Sigma: strain-level inference of genomes from metagenomic analysis for biosurveillance. *Bioinformatics* **31,** 170–177 (2015).
7. Van Dijk, L. R. *et al.* StrainGE: a toolkit to track and characterize low-abundance strains in complex microbial communities. *Genome biology* **23,** 1–27 (2022).
8. Reppell, M. & Novembre, J. Using pseudoalignment and base quality to accurately quantify microbial community composition. *PLoS computational biology* **14,** e1006096 (2018).
9. Luo, C. *et al.* ConStrains identifies microbial strains in metagenomic datasets. *Nature biotechnology* **33,** 1045–1052 (2015).
10. Smillie, C. S. *et al.* Strain tracking reveals the determinants of bacterial engraftment in the human gut following fecal microbiota transplantation. *Cell host & microbe* **23,** 229–240 (2018).
11. Segata, N. *et al.* Metagenomic microbial community profiling using unique clade-specific marker genes. *Nature methods* **9,** 811–814 (2012).
12. Truong, D. T., Tett, A., Pasolli, E., Huttenhower, C. & Segata, N. Microbial strain-level population structure and genetic diversity from metagenomes. *Genome research* **27,** 626–638 (2017).
13. Browning, S. R. & Browning, B. L. Haplotype phasing: existing methods and new developments. *Nature Reviews Genetics* **12,** 703–714 (2011).
14. Schaeffer, L., Pimentel, H., Bray, N., Melsted, P. & Pachter, L. Pseudoalignment for metagenomic read assignment. *Bioinformatics* **33,** 2082–2088 (2017).
15. Lee, Z. M.-P., Bussema III, C. & Schmidt, T. M. rrn DB: documenting the number of rRNA and tRNA genes in bacteria and archaea. *Nucleic acids research* **37,** D489–D493 (2009).
16. Smith, B. J., Li, X., Abate, A., Shi, Z. J. & Pollard, K. S. Scalable microbial strain inference in metagenomic data using StrainFacts. *bioRxiv* (2022).
17. Kucukelbir, A., Tran, D., Ranganath, R., Gelman, A. & Blei, D. M. Automatic differentiation variational inference. *Journal of machine learning research* (2017).
18. Hoffman, M. D., Blei, D. M., Wang, C. & Paisley, J. Stochastic variational inference. *Journal of Machine Learning Research* (2013).

19. Jaillard, M., Tournoud, M., Meynier, F. & Veyrieras, J.-B. Optimization of alignment-based methods for taxonomic binning of metagenomics reads. *Bioinformatics* **32,** 1779–1787 (2016).

20. Schirmer, M., D'Amore, R., Ijaz, U. Z., Hall, N. & Quince, C. Illumina error profiles: resolving fine-scale variation in metagenomic sequencing data. *BMC bioinformatics* **17,** 1–15 (2016).
