# Supplementary material for: Strain Tracking with Uncertainty Quantification
Source: bioRxiv. 2023 Jan 26:2023.01.25.525531. Preprint. [Version 1] doi: 10.1101/2023.01.25.525531 (PMC9900846; doi:10.1101/2023.01.25.525531)
Supplement: Supplementary file 2 [file media-2.pdf]

Supplemental Figures

Complete UMB Results

Here, we provide all figures akin to participant UMB18's results shown in the main text. Participant UMB30, whose samples yielded no *E. coli* calls by either method, is omitted.

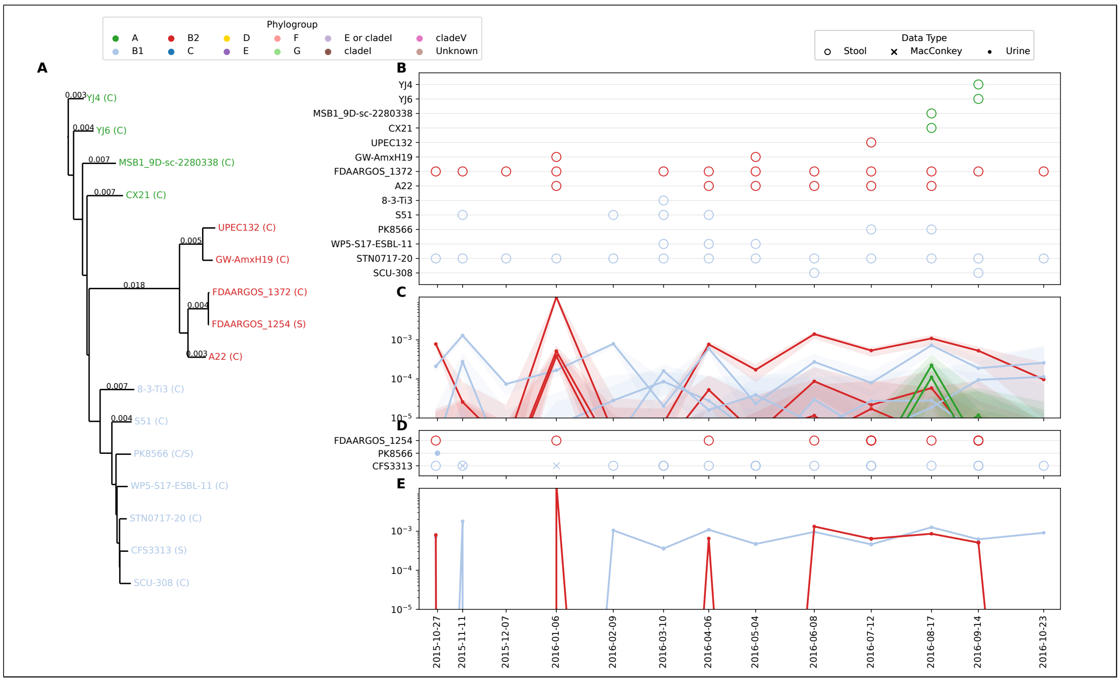

Supplemental Figure 1: Participant UMB01

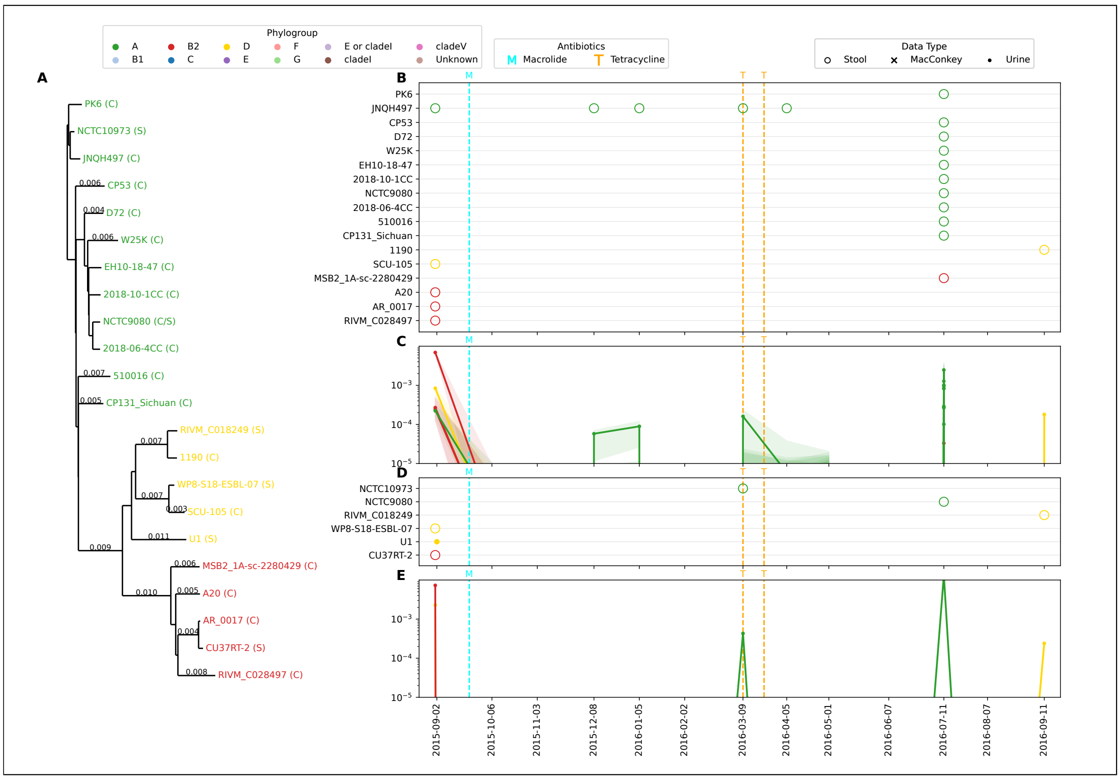

Supplemental Figure 2: Participant UMB02

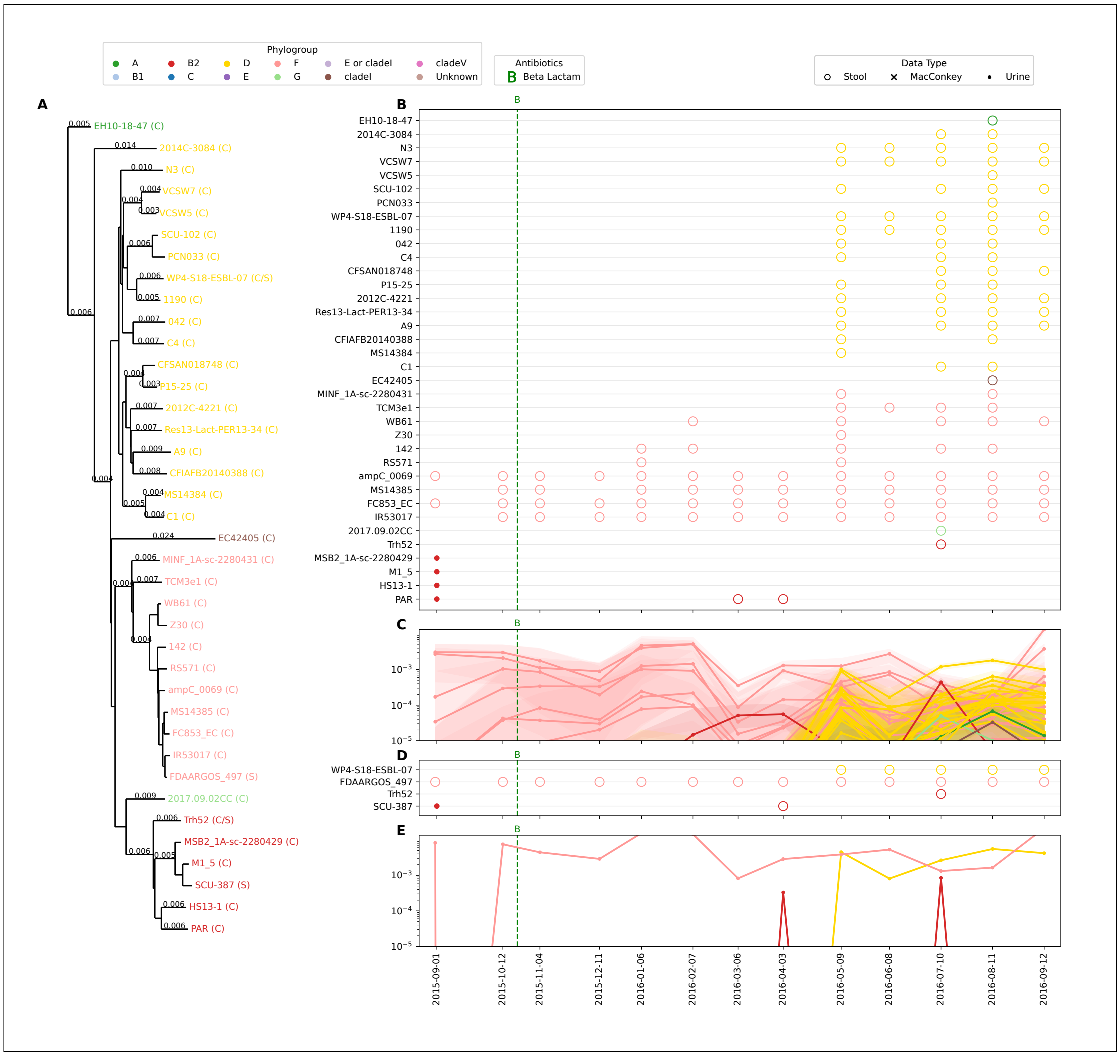

Supplemental Figure 3: Participant UMB03

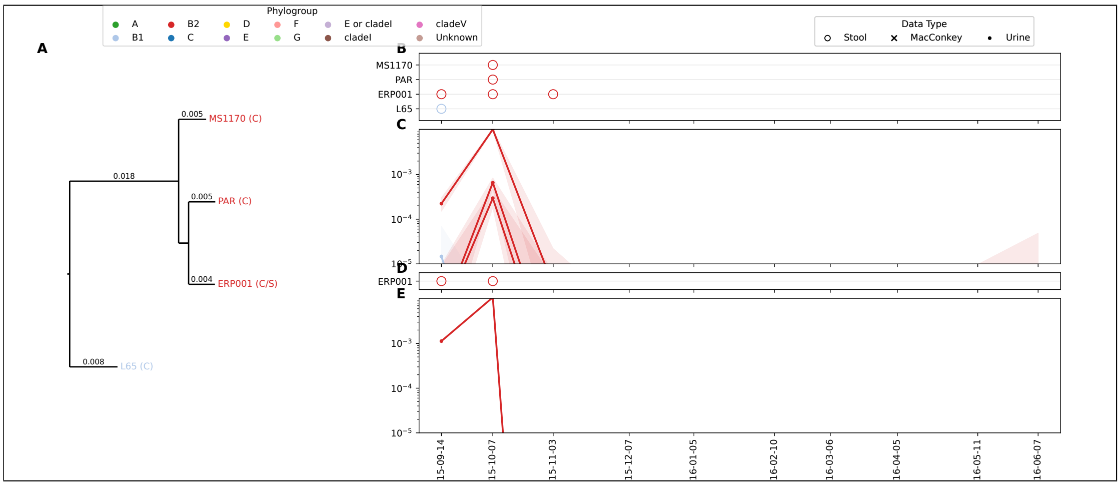

Supplemental Figure 4: Participant UMB04

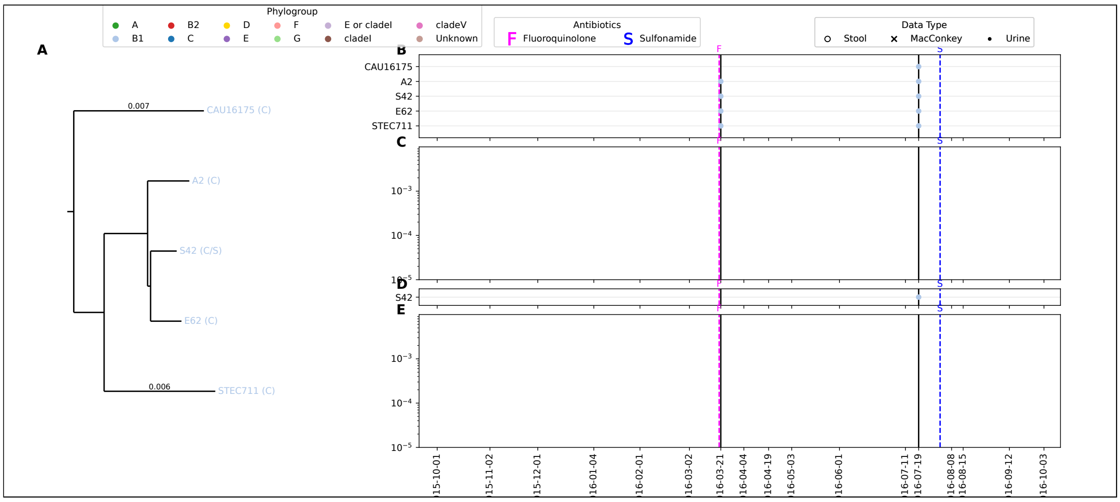

Supplemental Figure 5: Participant UMB05

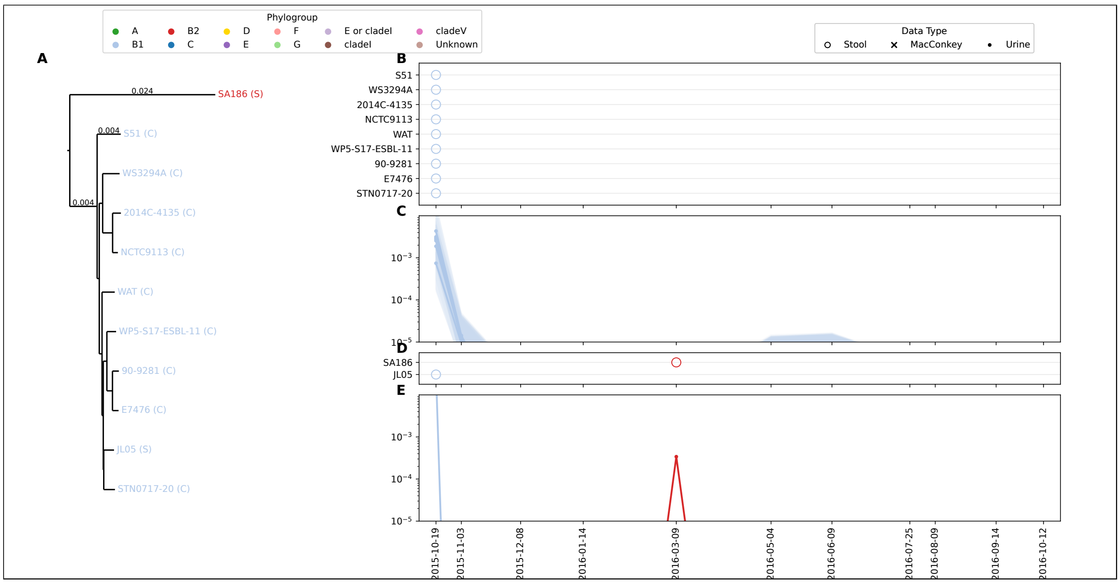

Supplemental Figure 6: Participant UMB06

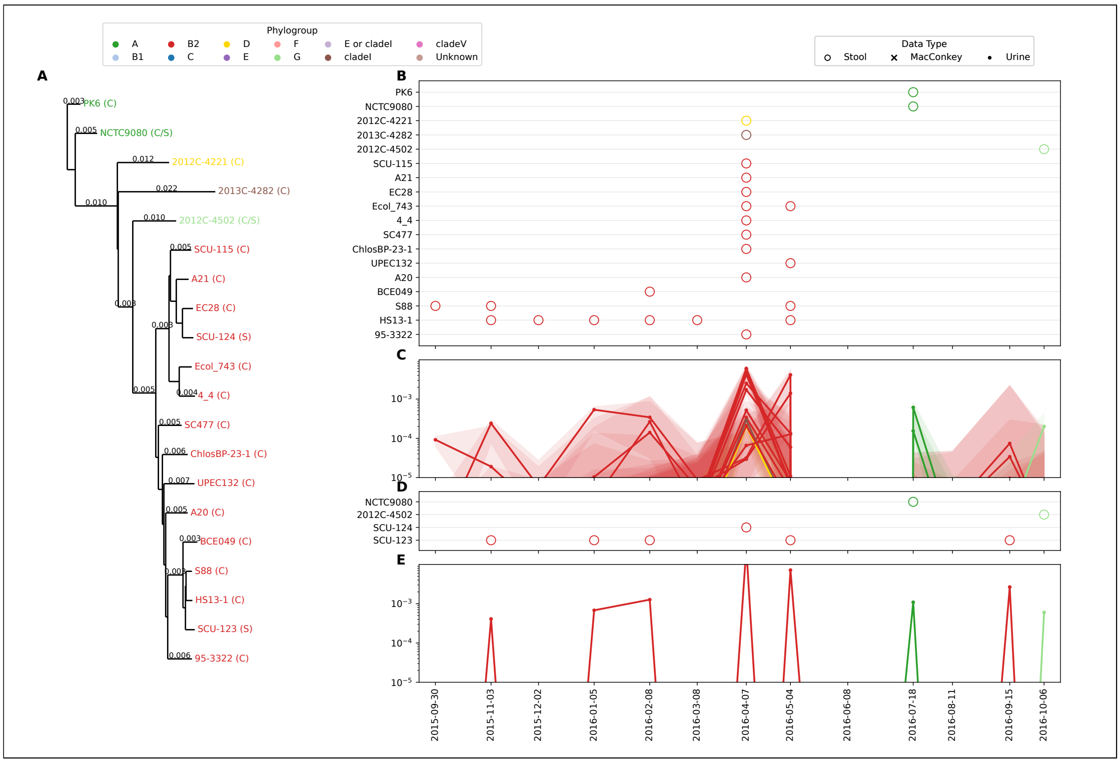

Supplemental Figure 7: Participant UMB07

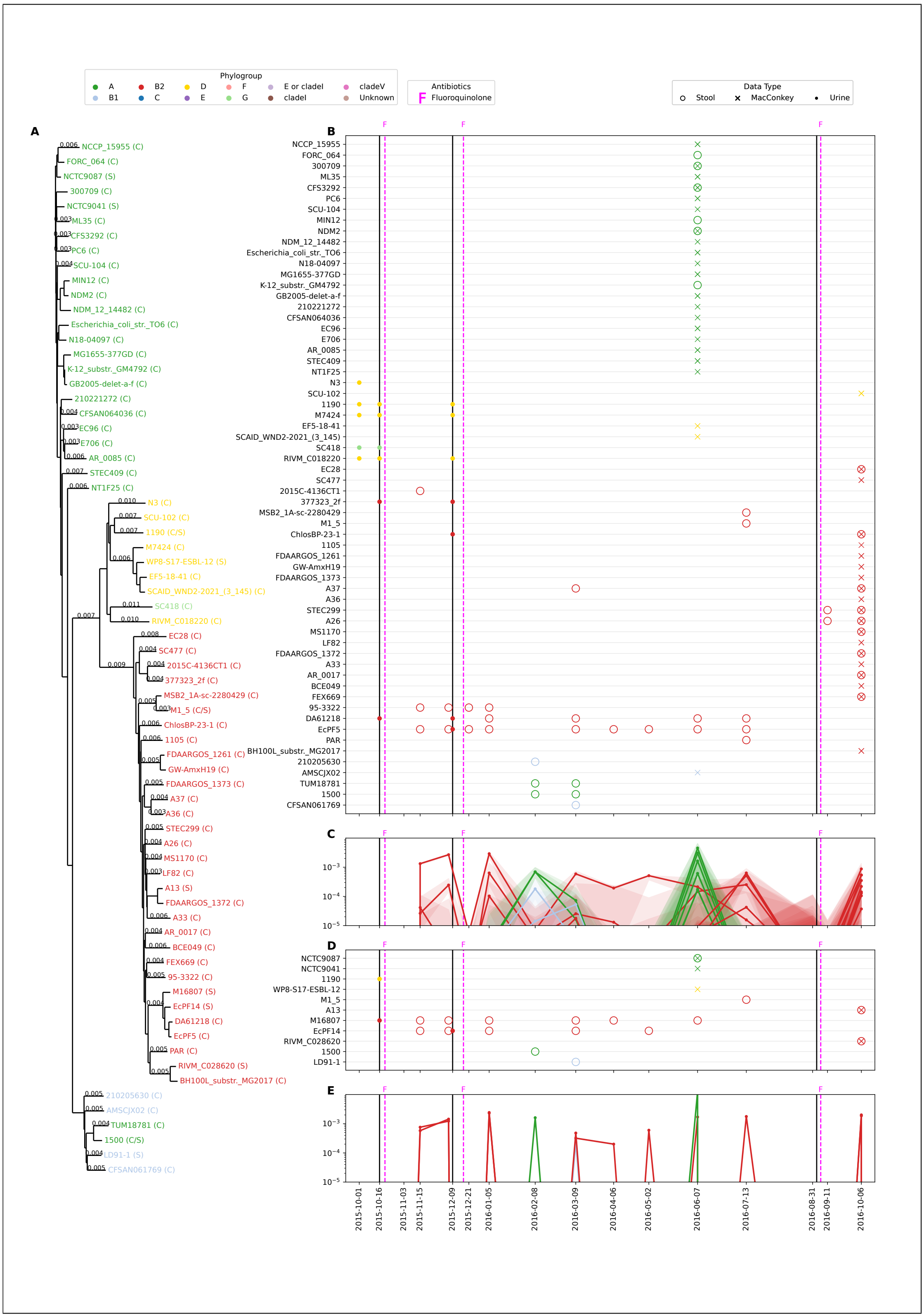

Supplemental Figure 8: Participant UMB08

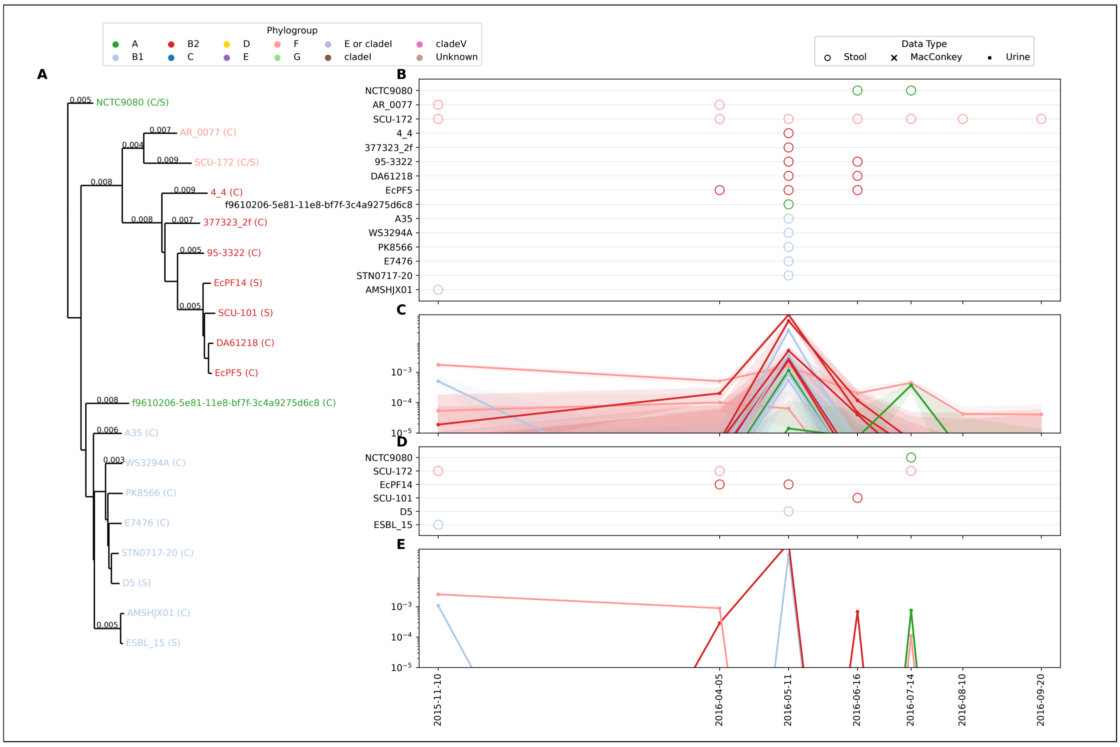

Supplemental Figure 9: Participant UMB09

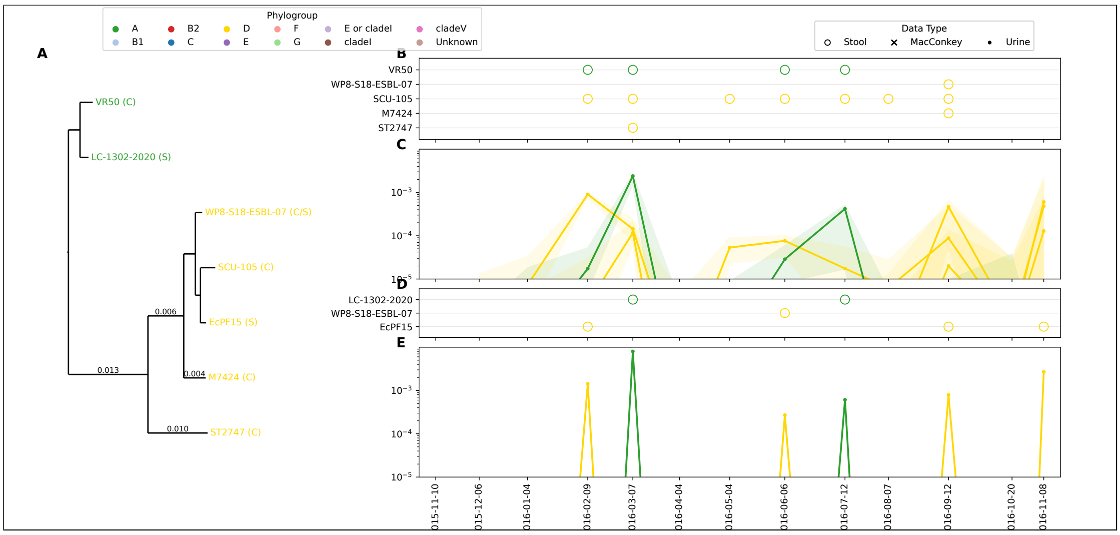

Supplemental Figure 10: Participant UMB10

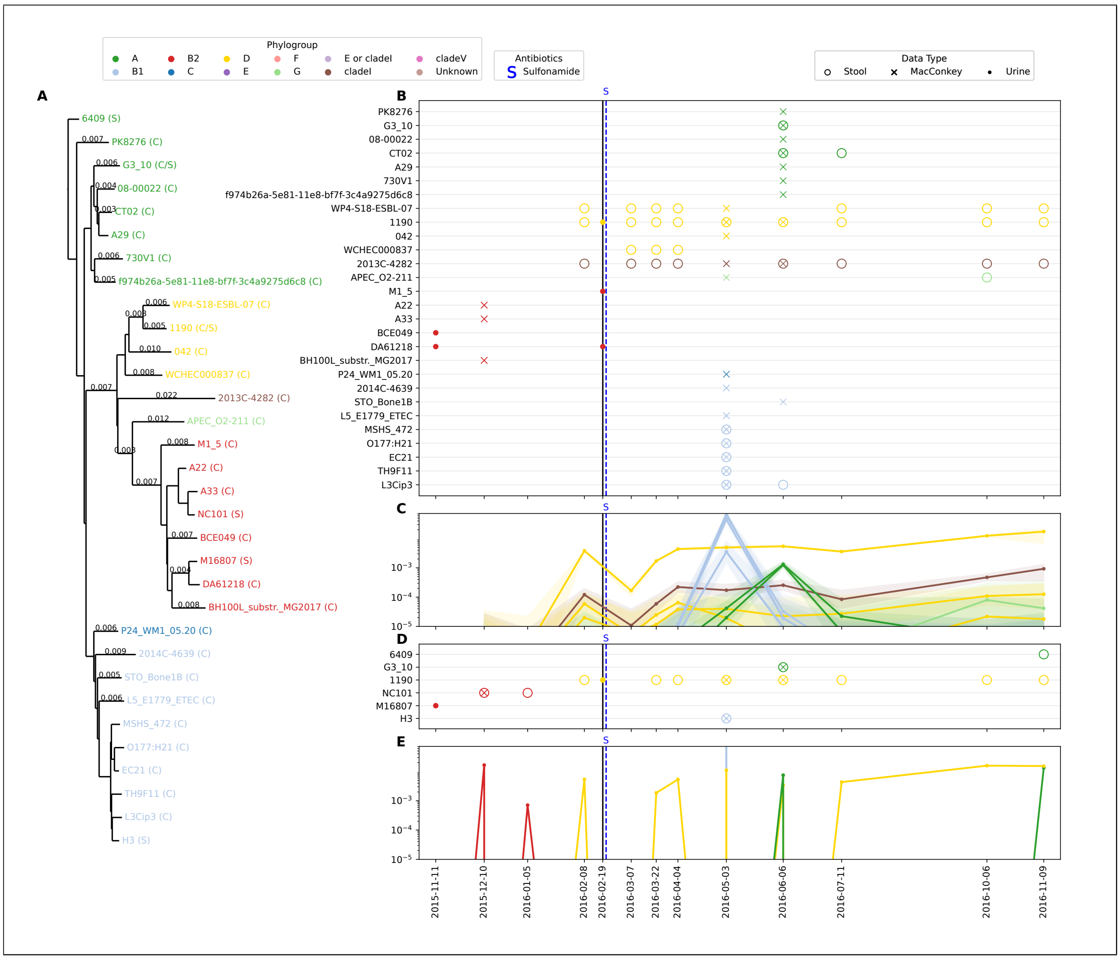

Supplemental Figure 11: Participant UMB11

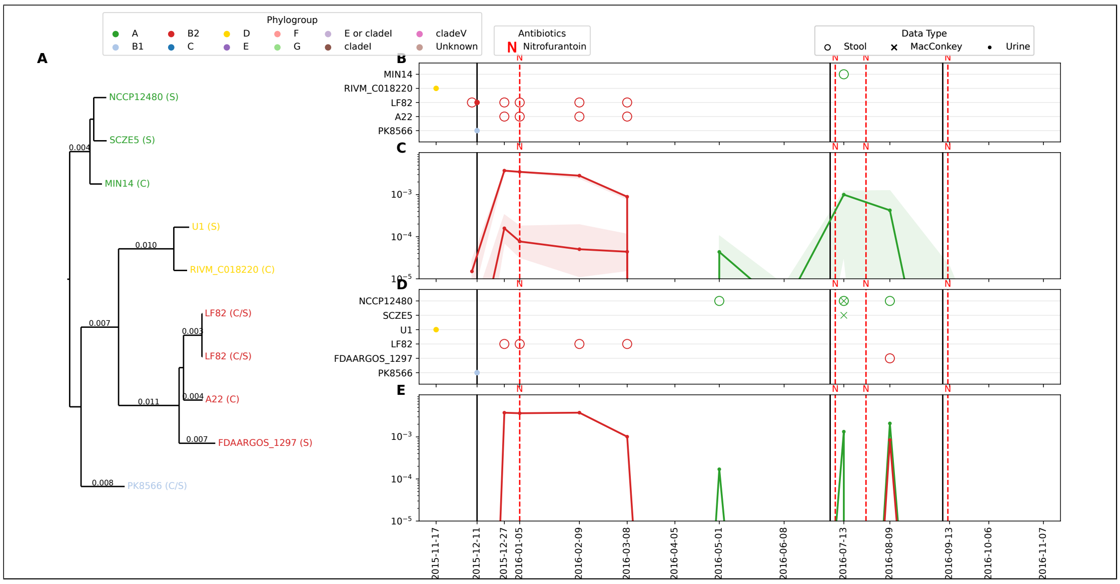

Supplemental Figure 12: Participant UMB12

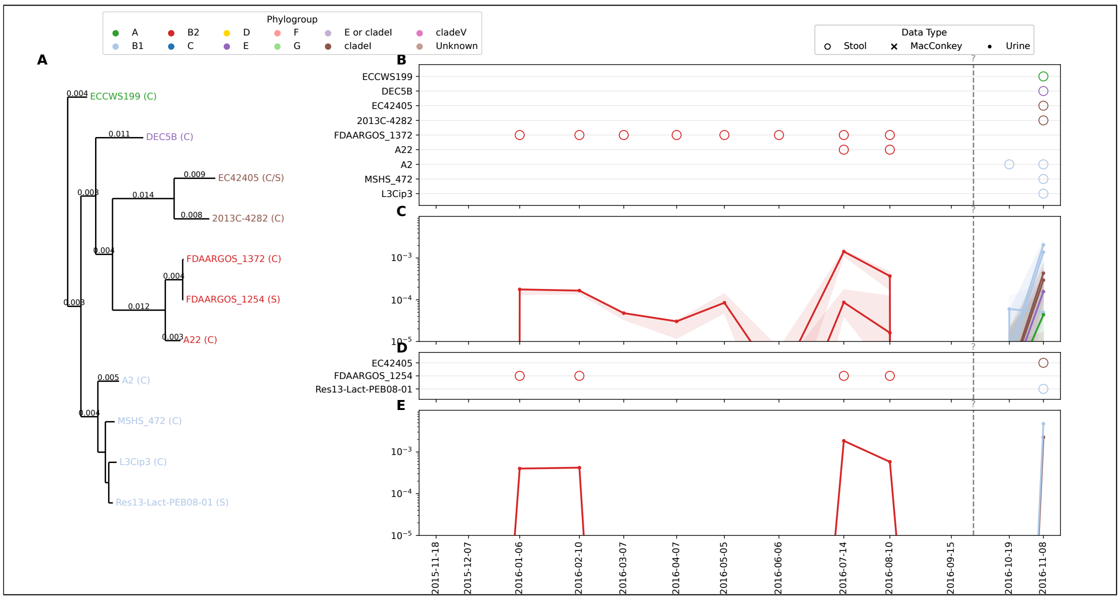

Supplemental Figure 13: Participant UMB13

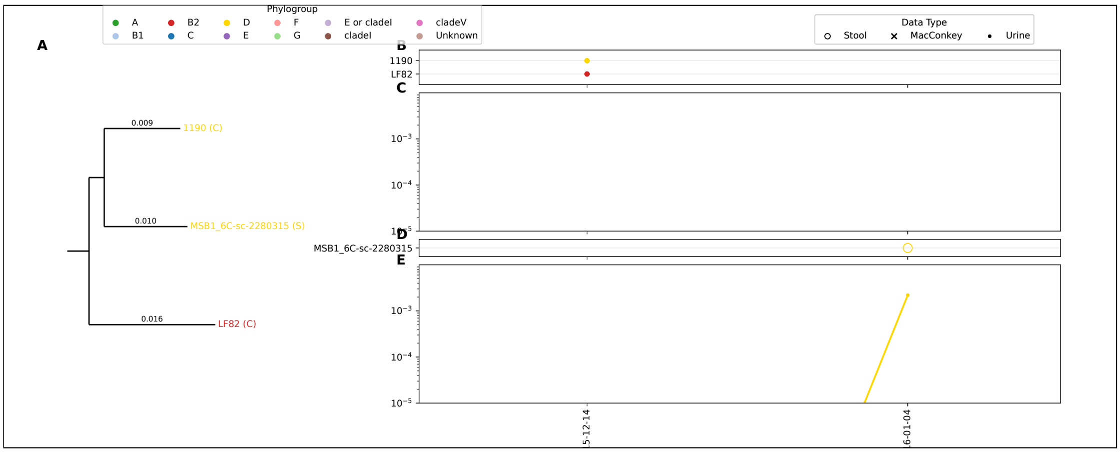

Supplemental Figure 14: Participant UMB14

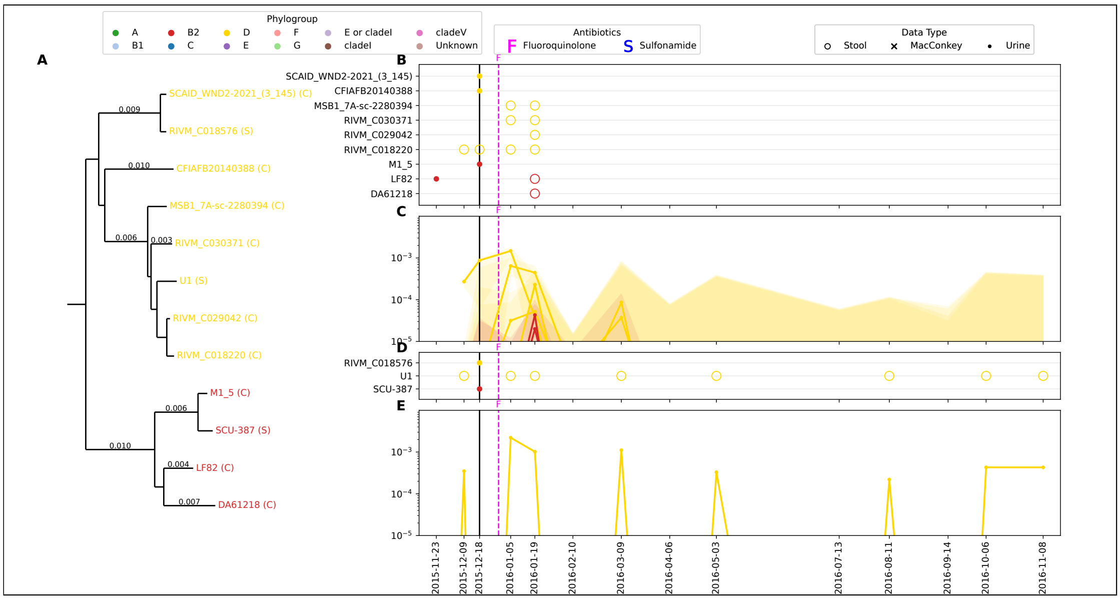

Supplemental Figure 15: Participant UMB15

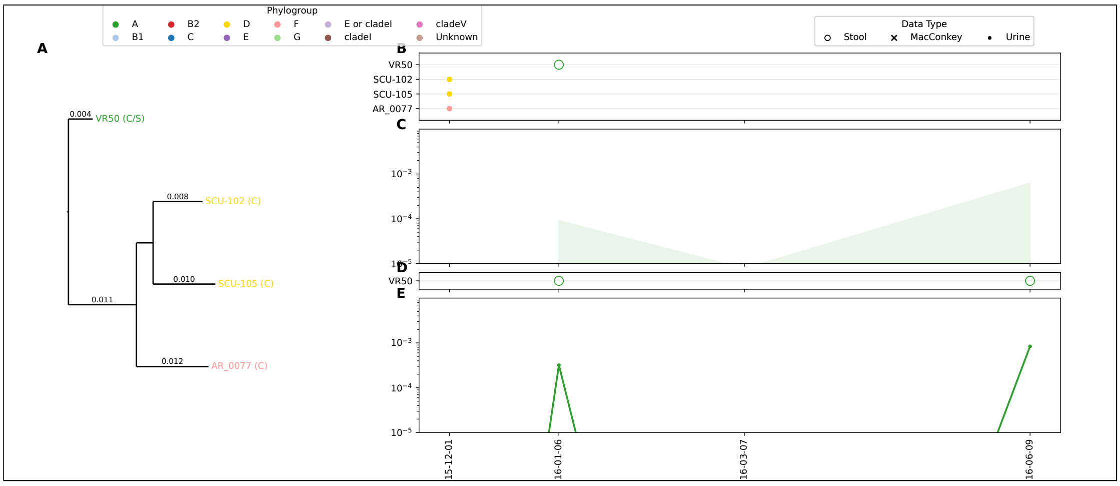

Supplemental Figure 16: Participant UMB16

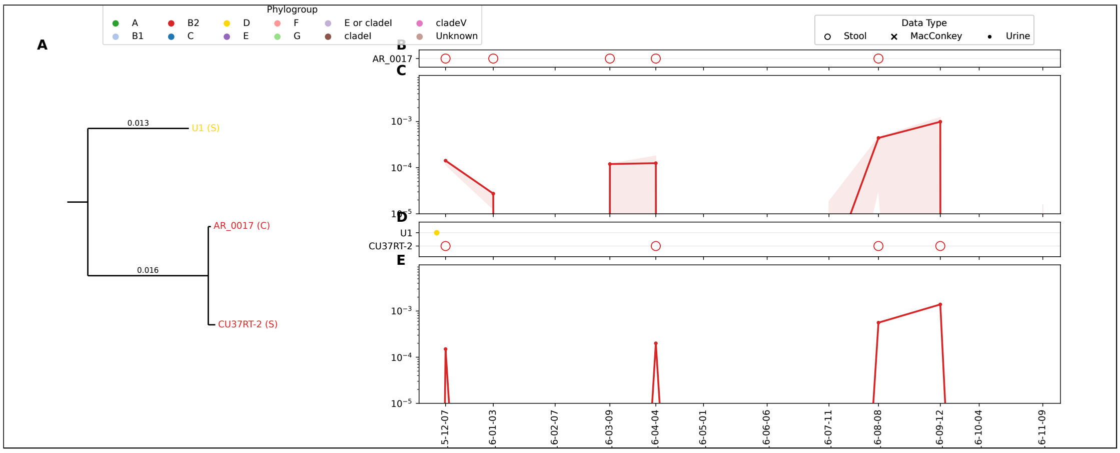

Supplemental Figure 17: Participant UMB17

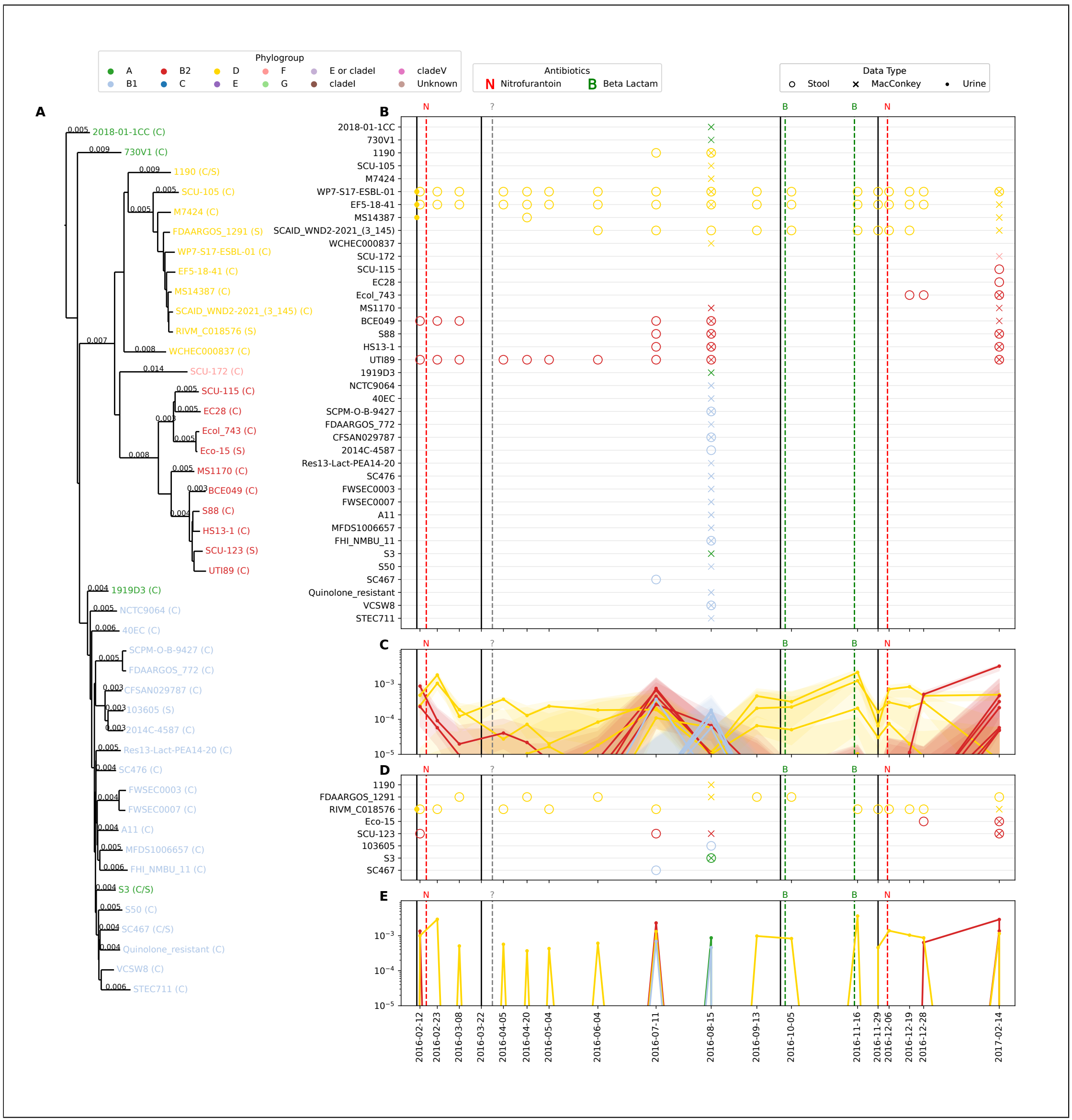

Supplemental Figure 18: Participant UMB18

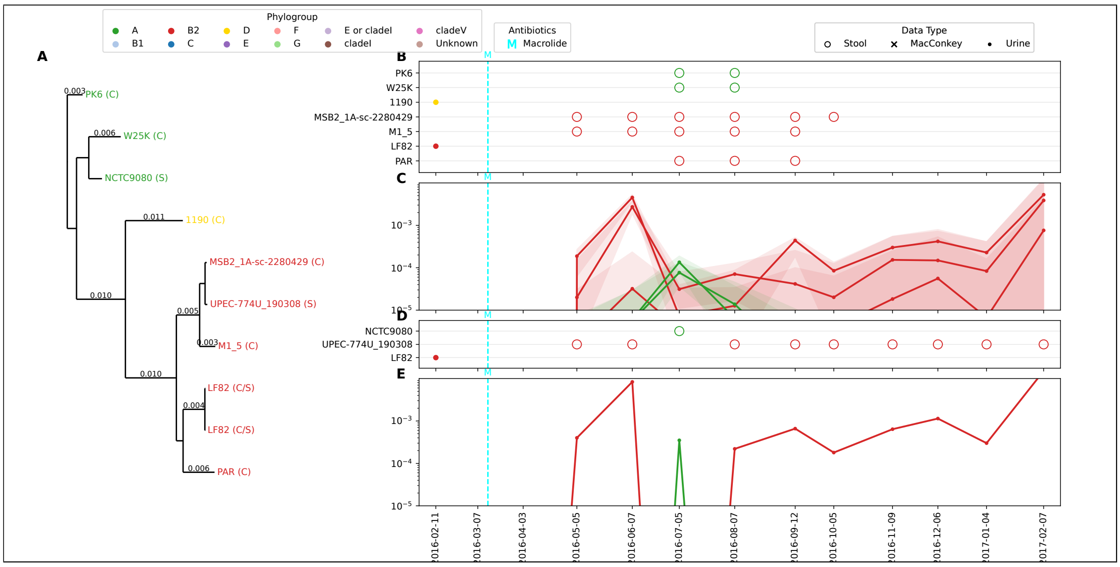

Supplemental Figure 19: Participant UMB19

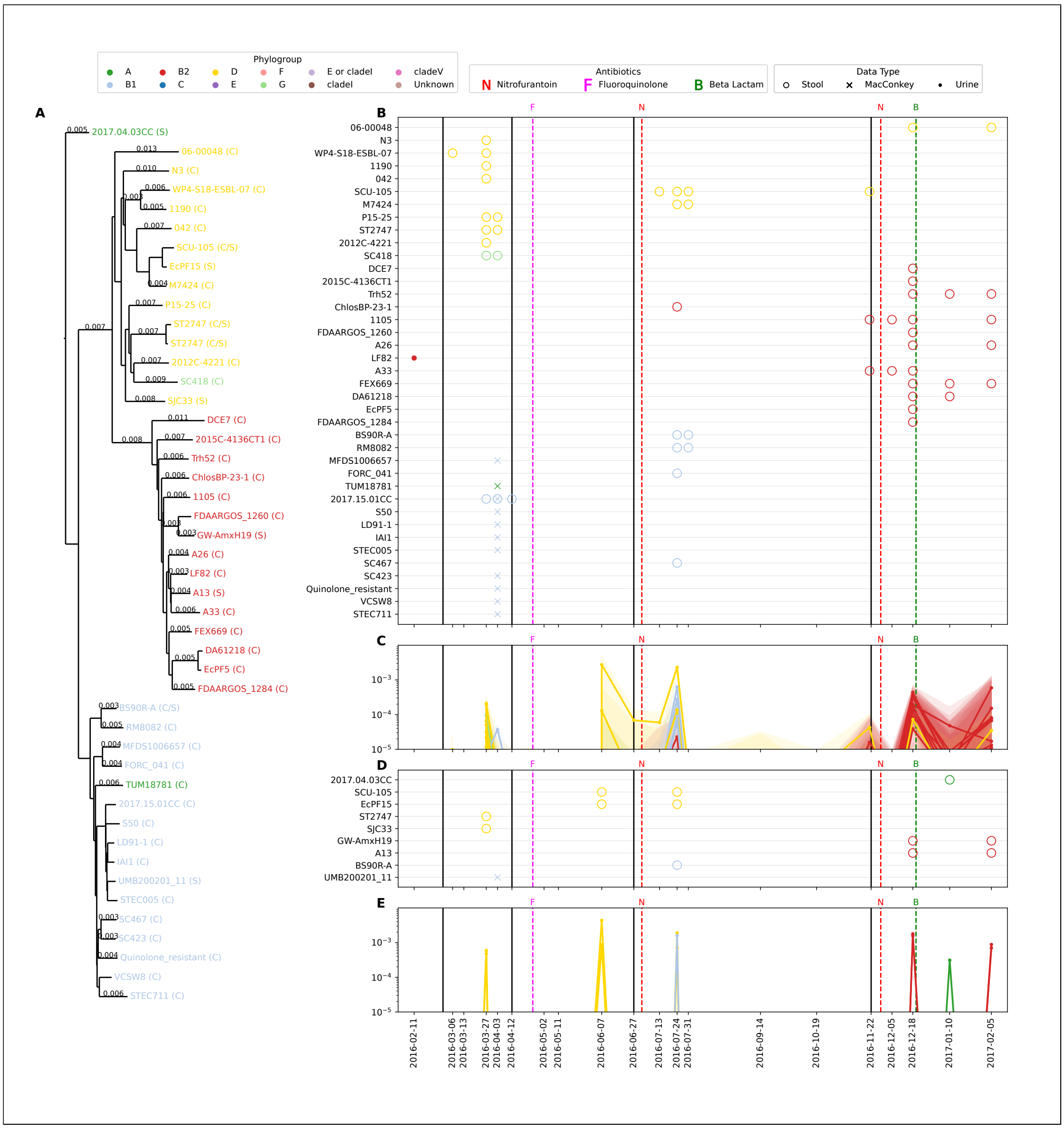

Supplemental Figure 20: Participant UMB20

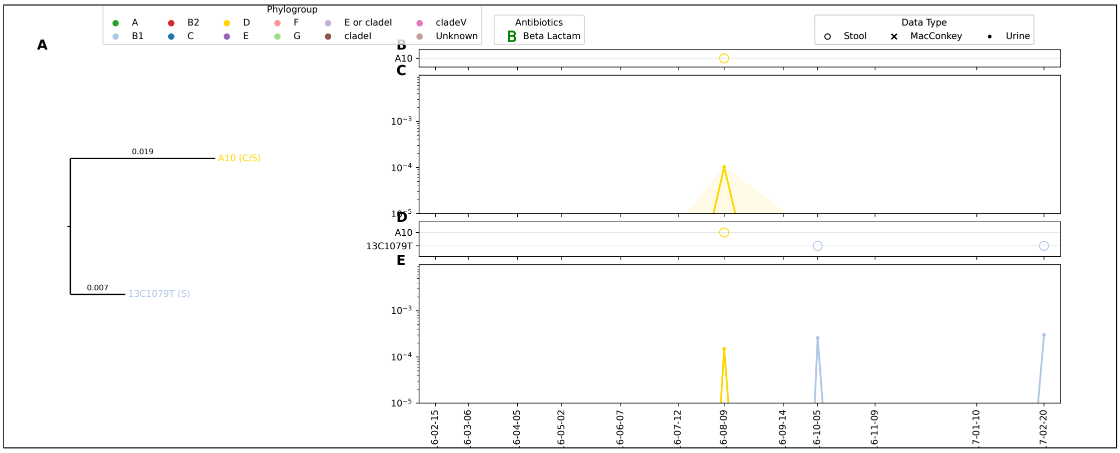

Supplemental Figure 21: Participant UMB21

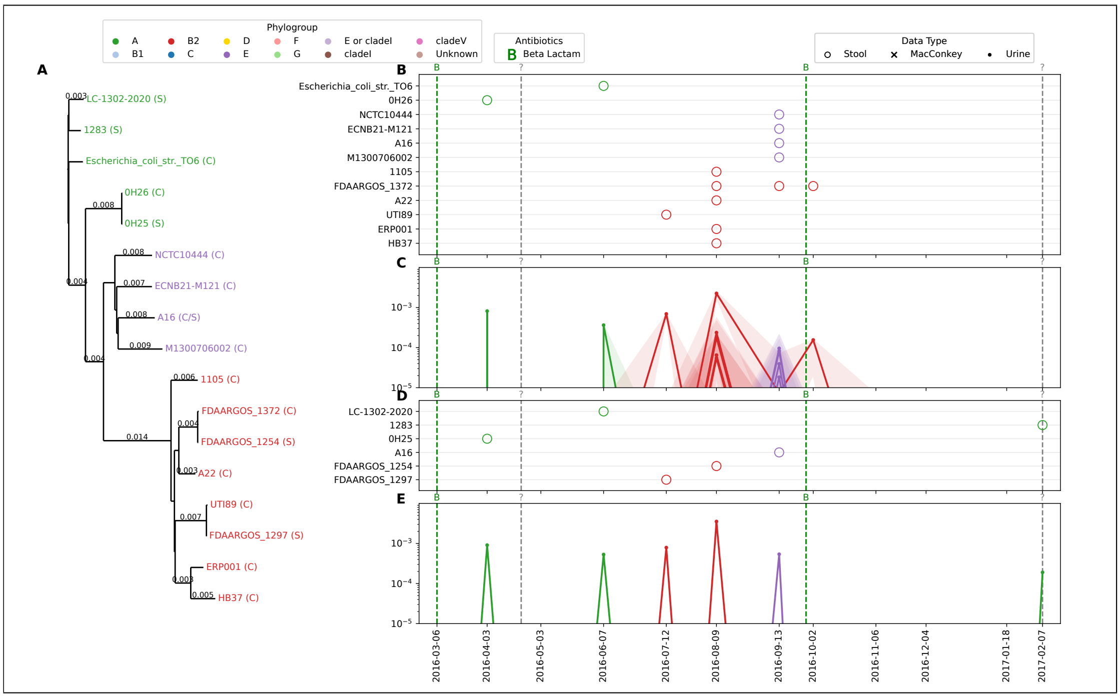

Supplemental Figure 22: Participant UMB22

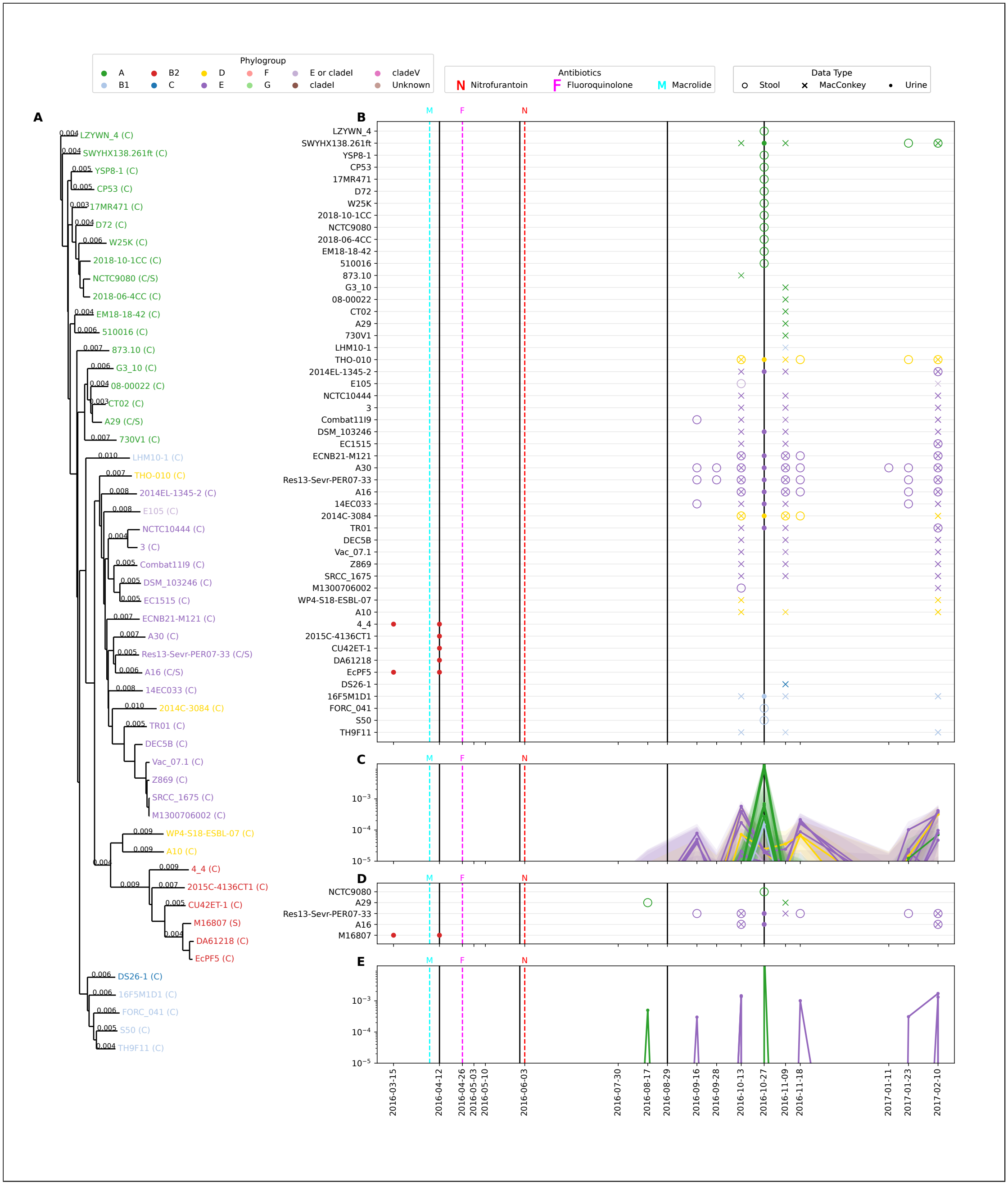

Supplemental Figure 23: Participant UMB23

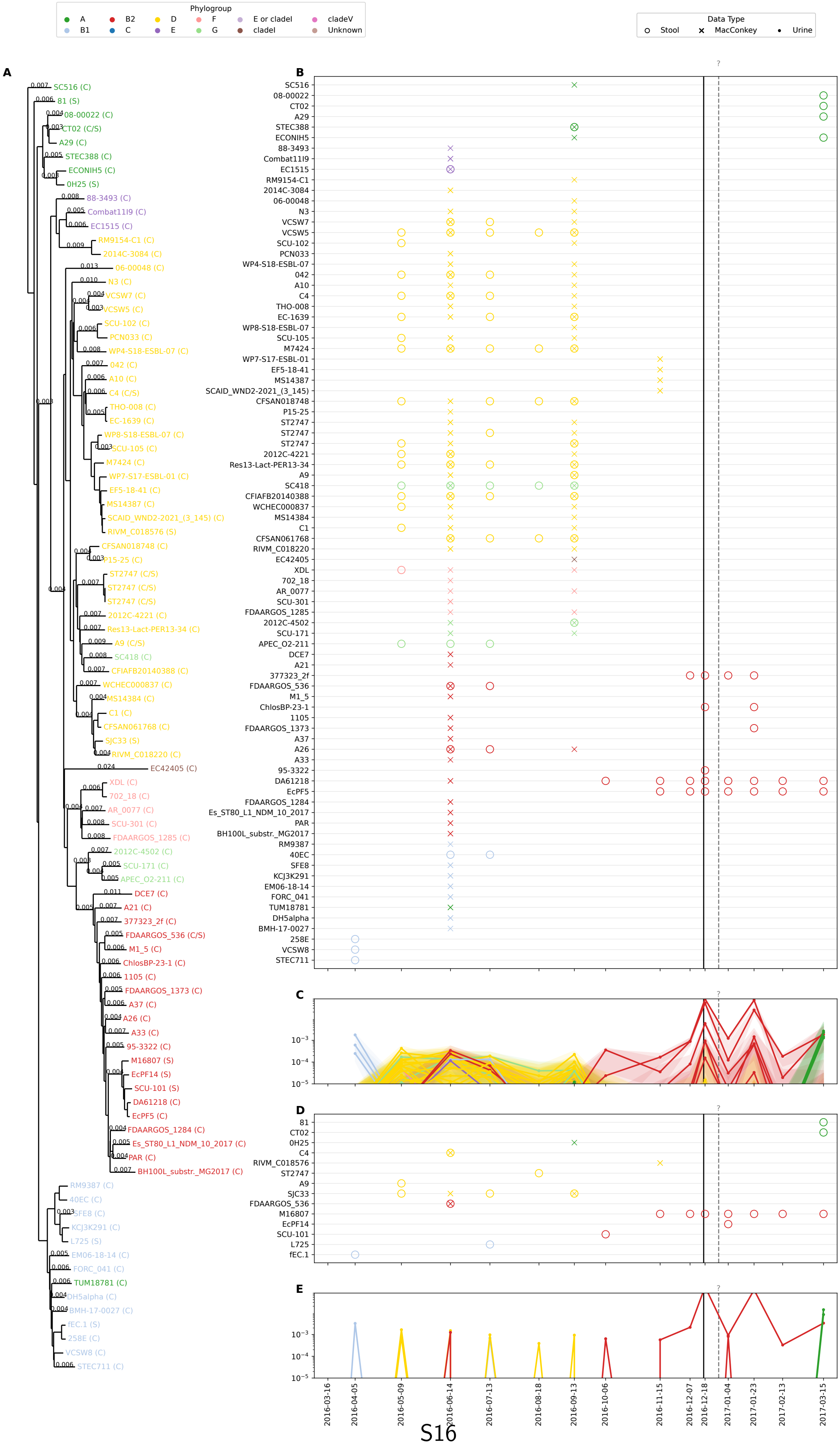
Phylogroup
A
B1
B2
C
D
E
F
G
E or cladeI
cladeI
cladeV
Unknown
Data Type
Stool
MacConkey
Urine
A
B
C
D
E
?
2016-03-16
2016-04-05
2016-05-09
2016-06-14
2016-07-13
2016-08-18
2016-09-13
2016-10-06
2016-11-15
2016-12-07
2016-12-18
2017-01-04
2017-01-23
2017-02-13
2017-03-15

S16

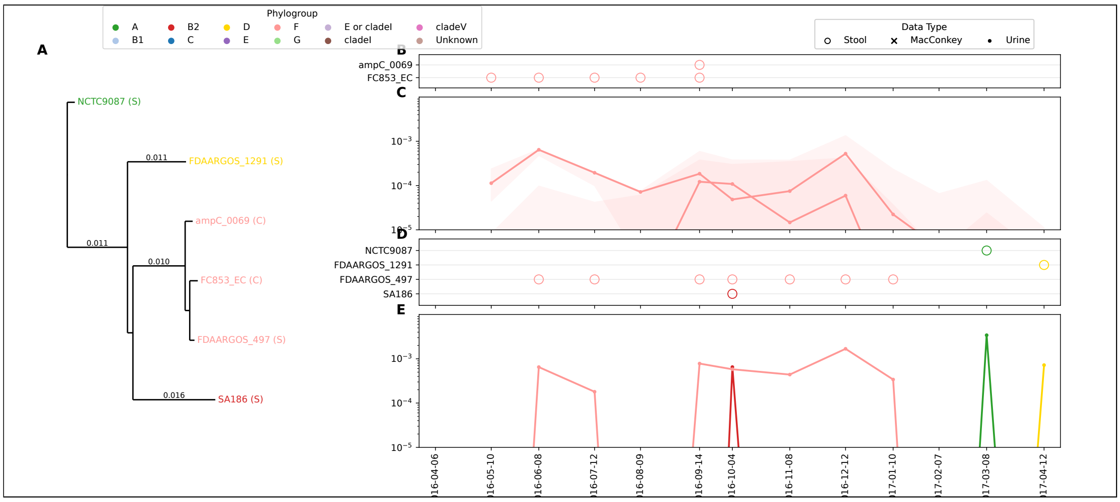

Supplemental Figure 25: Participant UMB25

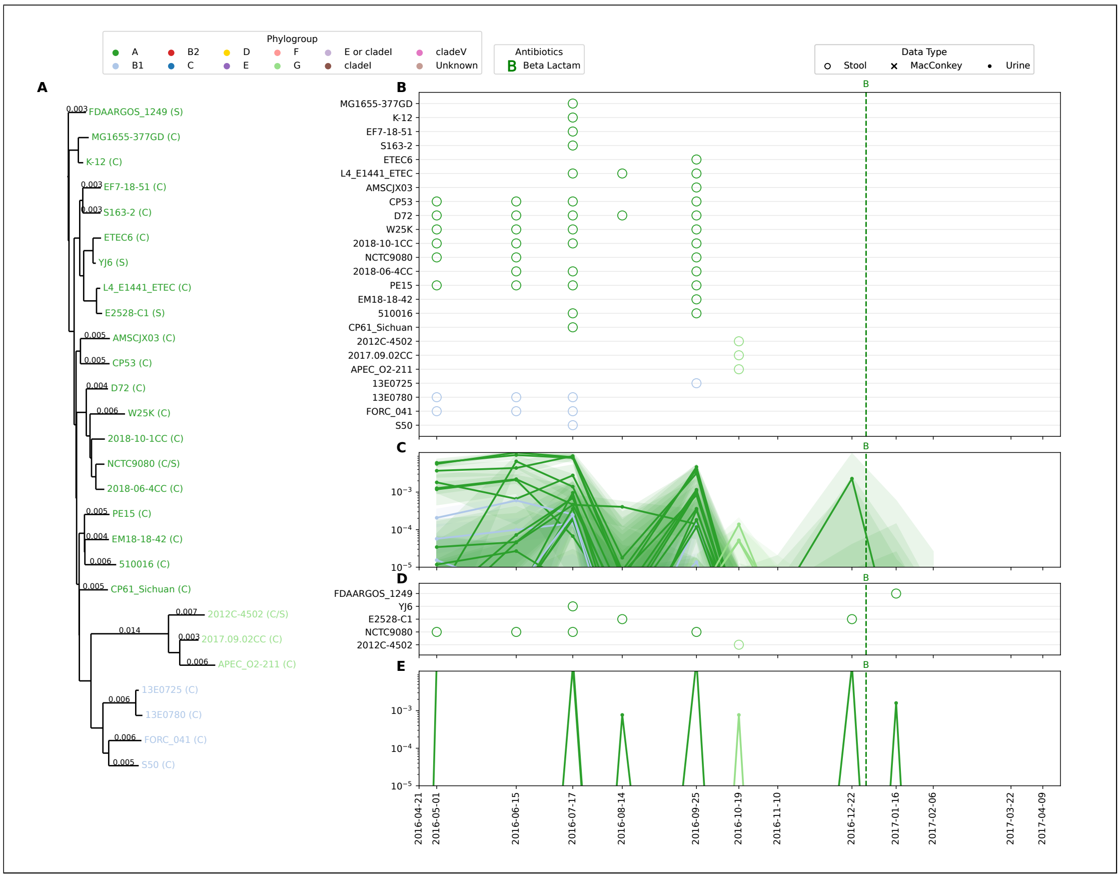

Supplemental Figure 26: Participant UMB26

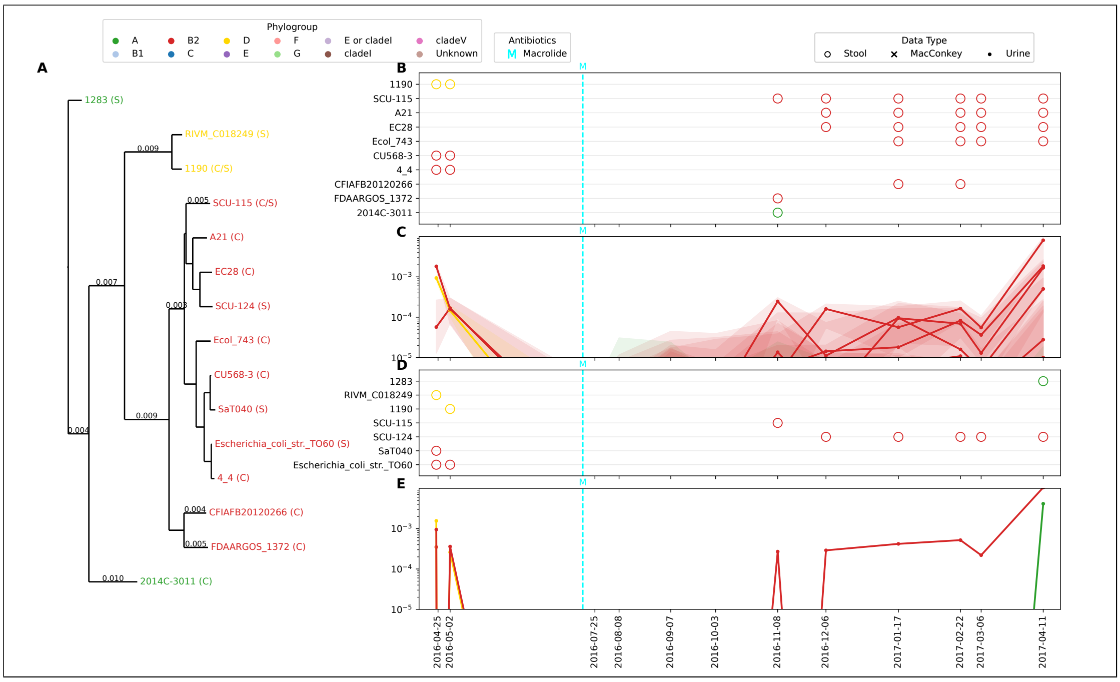

Supplemental Figure 27: Participant UMB27

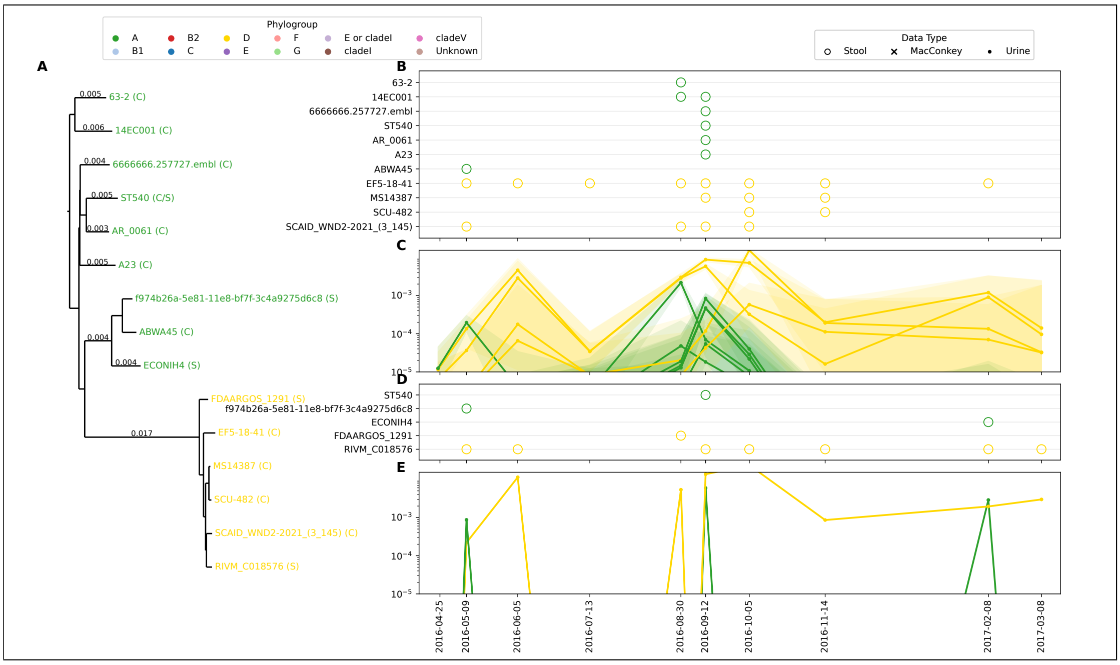

Supplemental Figure 28: Participant UMB28

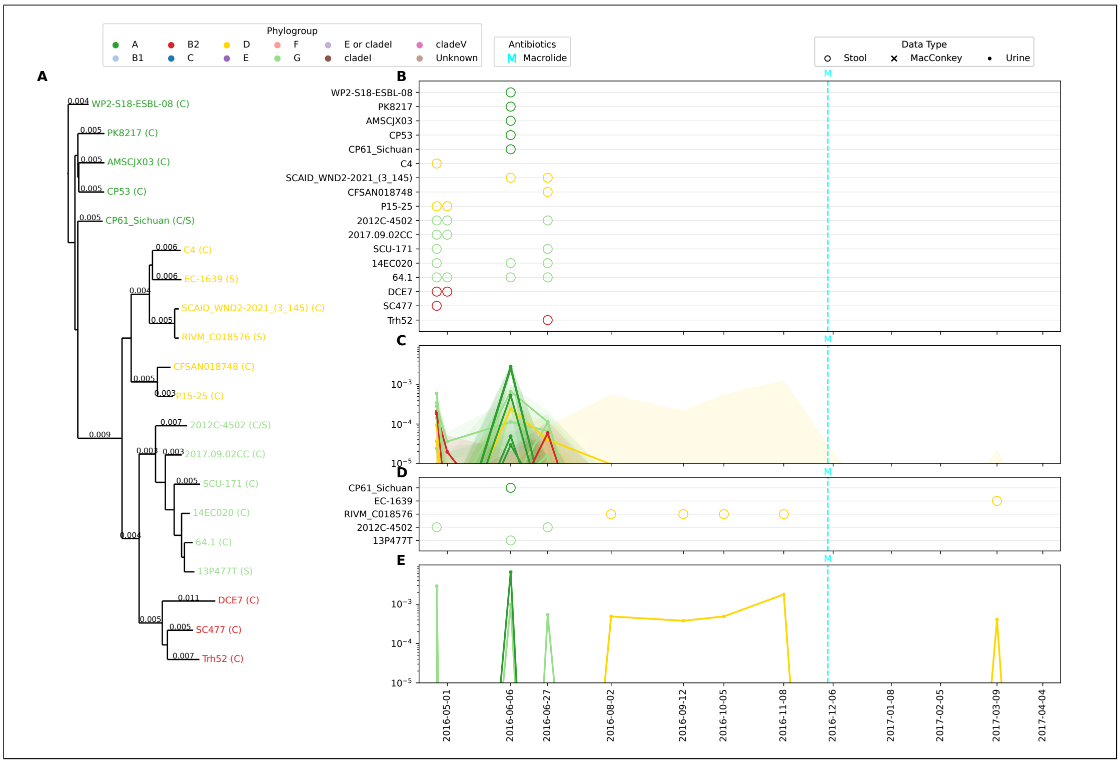

Supplemental Figure 29: Participant UMB29

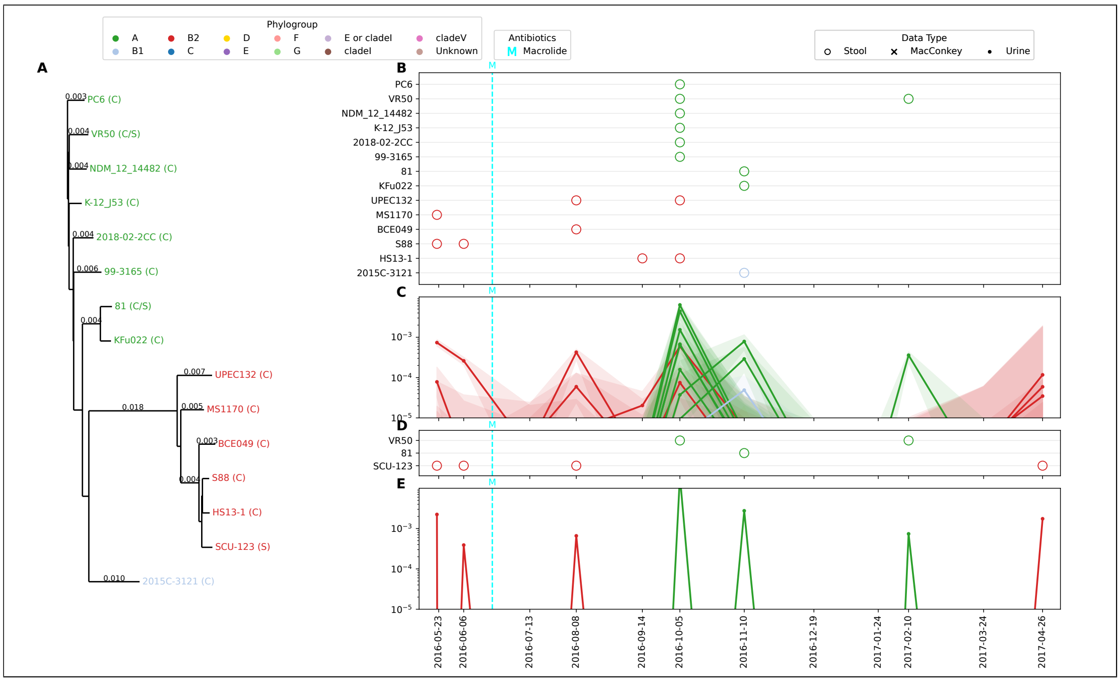

Supplemental Figure 30: Participant UMB31

Supplemental Tables

Fully Synthetic Experiment p-values

Diff. from ChronoStrain	# Simulated Reads				
	10^4	5×10^4	10^5	5×10^5	10^6
StrainGST	0.006	0.000	0.000	0.000	0.000
StrainEst	0.000	0.000	0.000	0.002	0.011

Supplemental Table 1: p-values from semisynthetic benchmark. Benjamini-Hochberg corrected p-values across all coverages and across all methods. Prior to the correction, each raw p-value measures the significance derived from a one-sided Wilcoxon test using $n = 10$ synthetic replicates.

Semisynthetic Experiment p-values

Diff. from ChronoStrain		Coverage				
		0.07x	0.15x	0.30x	0.75x	1.50x
StrainGST	TV Err.	0.002	0.002	0.002	0.002	1.000
	Recall	0.002	0.002	0.002	0.002	0.084
	Correl.	0.003	0.003	0.006	0.022	0.492
StrainEst	TV Err.	0.002	0.002	0.002	0.002	0.011
	Recall	0.002	0.002	0.002	0.002	0.010
	Correl.	0.003	0.003	0.003	0.003	0.013

Supplemental Table 2: p-values from semisynthetic benchmark. Separately for each metric, Benjamini-Hochberg correction was applied across all coverages and across all methods. Prior to the correction, each raw p-value measures the significance derived from a two-sided Wilcoxon test using $n = 10$ synthetic replicates.
